# An Understanding of Mechanism-Based Approaches for 1,3,4-Oxadiazole Scaffolds as Cytotoxic Agents and Enzyme Inhibitors

**DOI:** 10.3390/ph16020254

**Published:** 2023-02-07

**Authors:** Davinder Kumar, Navidha Aggarwal, Aakash Deep, Harsh Kumar, Hitesh Chopra, Rakesh Kumar Marwaha, Simona Cavalu

**Affiliations:** 1 Department of Pharmaceutical Sciences, Maharshi Dayanand University, Rohtak 124001, India; 2 MM College of Pharmacy, Maharishi Markandeshwar (Deemed to be University), Mullana, Ambala 133207, India; 3 Department of Pharmaceutical Sciences, Chaudhary Bansi Lal University, Bhiwani 127021, India; 4 Chitkara College of Pharmacy, Chitkara University, Rajpura 140401, India; 5 Faculty of Medicine and Pharmacy, University of Oradea, P-ta 1 Decembrie 10, 410087 Oradea, Romania

**Keywords:** 1,3,4-oxadiazole, synthesis, telomerase, HDAC, thymidylate synthase, anticancer potential

## Abstract

The world’s health system is plagued by cancer and a worldwide effort is underway to find new drugs to treat cancer. There has been a significant improvement in understanding the pathogenesis of cancer, but it remains one of the leading causes of death. The imperative 1,3,4-oxadiazole scaffold possesses a wide variety of biological activities, particularly for cancer treatment. In the development of novel 1,3,4-oxadiazole-based drugs, structural modifications are important to ensure high cytotoxicity towards malignant cells. These structural modification strategies have shown promising results when combined with outstanding oxadiazole scaffolds, which selectively interact with nucleic acids, enzymes, and globular proteins. A variety of mechanisms, such as the inhibition of growth factors, enzymes, and kinases, contribute to their antiproliferative effects. The activity of different 1,3,4-oxadiazole conjugates were tested on the different cell lines of different types of cancer. It is demonstrated that 1,3,4-oxadiazole hybridization with other anticancer pharmacophores have different mechanisms of action by targeting various enzymes (thymidylate synthase, HDAC, topoisomerase II, telomerase, thymidine phosphorylase) and many of the proteins that contribute to cancer cell proliferation. The focus of this review is to highlight the anticancer potential, molecular docking, and SAR studies of 1,3,4-oxadiazole derivatives by inhibiting specific cancer biological targets, such as inhibiting telomerase activity, HDAC, thymidylate synthase, and the thymidine phosphorylase enzyme. The purpose of this review is to summarize recent developments and discoveries in the field of anticancer drugs using 1,3,4-oxadiazoles.

## 1. Introduction

Humankind faces one of the most challenging of public health challenges, in the form of cancer. The development of cancer involves the uncontrollable division of abnormal cells that can infiltrate and destroy normal tissues in the body. High morbidity and mortality rates characterize the disease; after cardiovascular disease, it is the second leading cause of death in many countries. In order to reduce cancer death rates worldwide, chemotherapy is currently the main treatment modality for cancer, whether used alone or combined with surgery and radiotherapy [1].

**Figure 1:**
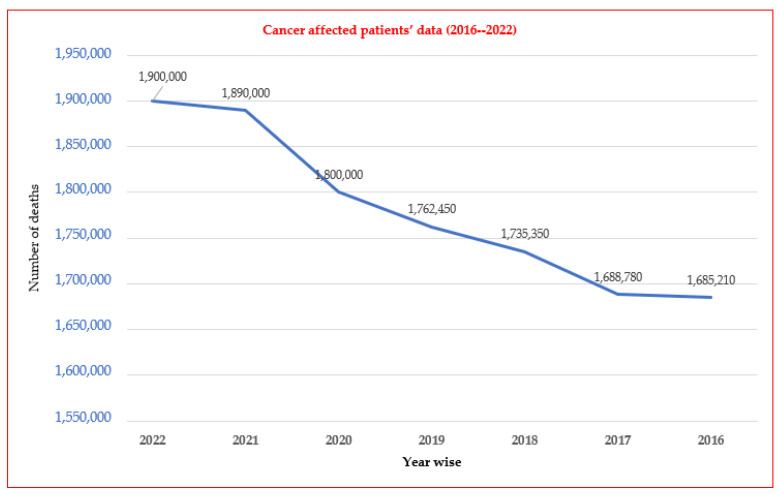
Cancer affected patients per year [4].

The American Cancer Society (ACS) provide data on cancer mortality in the United States. In 2016, there were an estimated 1,630,730 new cancer cases, and 589,390 cancer-related deaths in the United States. In 2017, due to advances in early detection and treatment, and changes in population age distribution, the ACS expects that those estimates will increase by about 8%.. Figure 1 represents the significant share of individuals affected by cancer each year [2,3].

**Figure 2:**
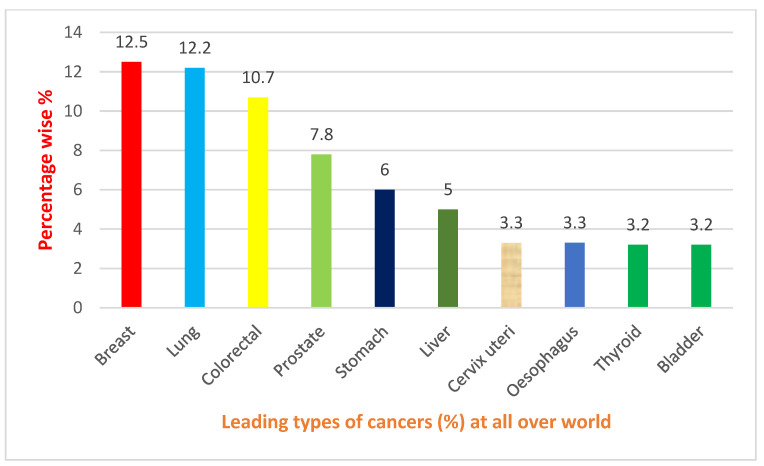
Leading types of cancers worldwide.

In 2018, about 9.6 million deaths and 19.1 million cancer diagnoses were reported globally, according to the International Agency for Research on Cancer (IARC). The North American Association of Central Cancer Registries (NAACCR), a professional cancer registry organization, has compiled data on the main cancers that led to thousands of deaths in the United States in 2021. Ten types of cancer are identified by NAACCR and IARC as the world’s leading cause of death (Figure 2). The treatment of cancer is a multidisciplinary effort involving physicians, oncologists, and surgeons and there are many different treatment strategies available worldwide. The treatment strategy for cancer depends on the type, stage, and the location of the cancer. This is determined by several factors including: age (children may need a different treatment plan to adults); how far the disease has progressed (stage); the stage of growth at the time surgery is performed (what has happened since diagnosis); how advanced the disease is at the time of treatment; and whether it can be cured or not (curable vs. incurable).The treatment of cancer has become a highly specialized technology, which involves diverse approaches, such as radiation therapy and biotechnology. In addition, novel methods of diagnosis and external therapies such as surgery, chemotherapy, radiotherapy, targeted therapies, and immune therapies such as interferon, are currently on going therapies in cancer hospitals and research laboratories across the world [4].

In recent years, research in the synthetic and semi-synthetic fields has become more prevalent, because of cancer’s life-threatening nature. Therefore, the need for new cancer therapies is increasing as researchers and clinicians try to find solutions to the problem of drug shortages [5,6].

**Figure 3:**
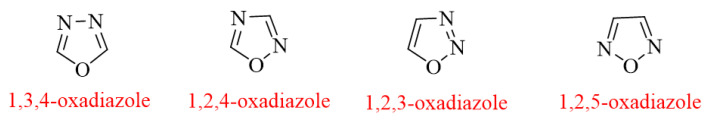
The isomers of oxadiazole.

Heterocyclic compounds, i.e., 5-membered and 6-membered rings, or fused ring systems, play an imperative role in the finding and progress of new drug molecules with the highest potency and lower toxicity [7]. Despite this, rings containing an N, O, or S atom have become a magnet in the field of synthetic chemistry in order to develop new medicinal compounds because of their huge therapeutic potentials. Research investigating different types of heterocyclic moieties, i.e., H. pyrazoles, tetrazoles, oxadiazoles, thiadiazoles, triazoles, etc., has shown attraction in recent years [8]. The 1,3,4-oxadiazole has four different isomers (Figure 3) and among them, the 1,2,3-isomer has shown instability due to the formation of diazoketone tautomers (ring opening) [9]. This heterocyclic ring was synthesized for the first time by Ainsworth in 1965, by the thermal decomposition reaction of hydrazine. Oxadiazole moiety with the formula C_2_H_2_ON_2_, 70.05 g/mol molecular weight and water soluble [10].

The oxadiazole skeleton is also referred to as Azoximes, Diazoxol, Furadiazole, Biozol, Furoxanes, and Oxybiazole. The oxadiazole ring is thermostable due to its resonance energy near 167.4 kJ/mol, which increases its thermal stability with substitution at the 2nd position [11].

**Figure 4:**
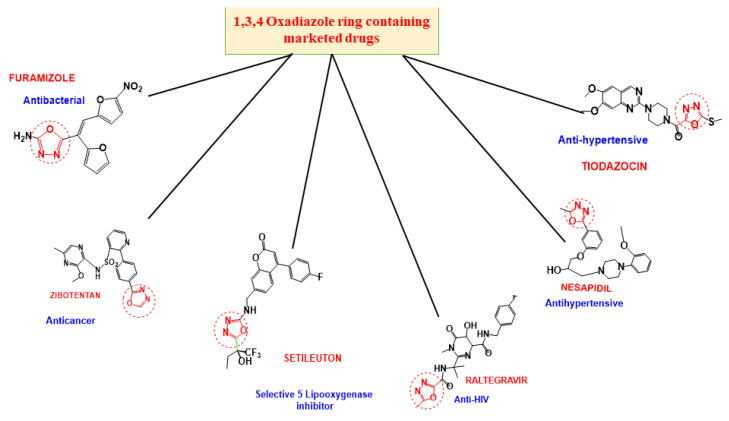
The commercial drugs of 1,3,4-oxadiazole ring.

Due of their ability to form hydrogen bonds with the receptor site, oxadiazole or its isomers are of considerable interest to chemical, medical, and pharmaceutical research for the development of innovative drugs. Further, this molecular ring is commercially available in many important drugs, e.g., Furamizole, with potent antibacterial activity; Nesapidil, with antiarrhythmic activity; Raltegravir, as an antiviral drug; Tiodazosin, as an antihypertensive agent; and the preferred derivative of the FDA-approved anticancer drug, Zibotentan (Figure 4) [12,13].

The 1,3,4-oxadiazole has been a well-known pharmacophore for about 85 years and is in high demand in numerous biological and chemical fields [14]. Due to the presence of an additional heteroatom in the ring, and its inductive effect, oxadiazole shows very weak basic properties. Because nitrogen atoms (=N-) are present in the oxadiazole ring, it behaves as a conjugated diene, thus reducing its aromaticity [15].

**Figure 5:**
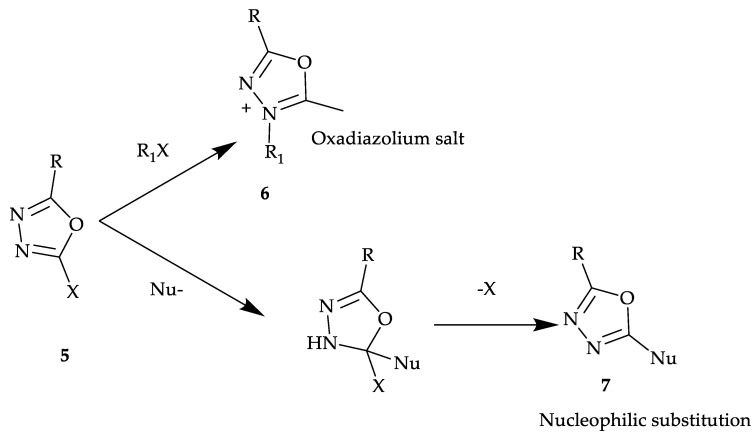
Nucleophilic substitutions.

Due to the lower electron cloud density in the oxadiazole moiety, electrophilic reactions at carbon atoms are difficult, resulting in 1,3,4-oxadiazolium salts. However, nucleophilic substitution reactions proceed in halogenated oxadiazole by replacing the halogen atom, which is illustrated in Figure 5 [16,17,18].

## 2. Various Synthetic Approaches for 1,3,4-Oxadiazole Derivatives (Figure 6)

**Scheme 1:**
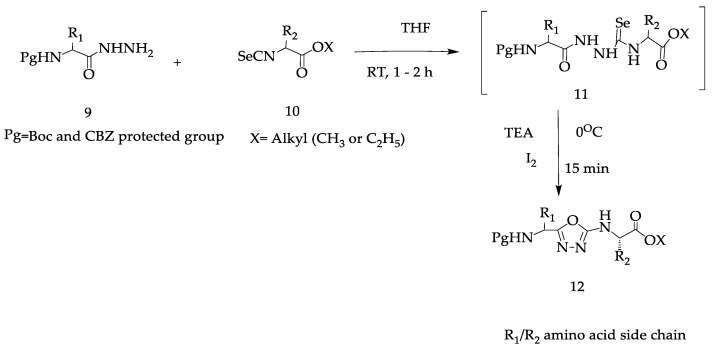
Synthetic route of 2-amino-1,3,4-oxadiazole derivatives.

L. Santhosh et al. synthesized a new series of 2-amino-1,3,4-oxadiazole linked peptidomimetics derivatives (Scheme 1) by one-pot synthesis [19]. This method involves the reaction between different Boc and Cbz-Nα-protected amino acid hydrazides with isoselenocyanato esters via cyclodeselenization, in the presence of THF and TEA at optimized temperature ranges, which derived selenosemicarbazide (**11**) as intermediates for 2-amino-1,3,4-oxadiazoles derivatives formation as end product (**12**). This kind of reaction produces high percentage yields in limited time, which is commercially cheap with safer conditions. Another convenient approach for 2-amino-1,3,4-oxadiazole conjugates (**15**) was prepared by Nelo R. Rivera et al. from acylthiosemicarbazide (**14**) through cyclization, followed by different acid chlorides (**13**) with thiosemicarbazide in THF [20].

**Figure 6:**
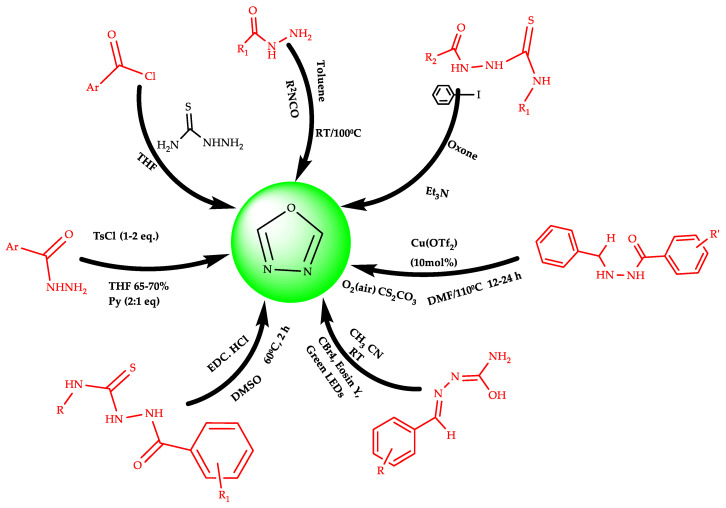
Various synthetic approaches for 1,3,4-oxadiazole derivatives.

**Scheme 2:**
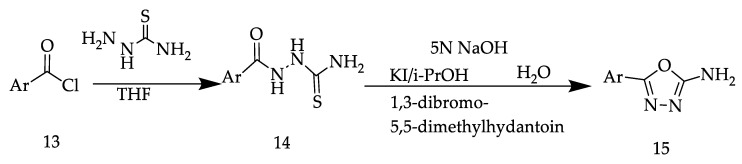
Synthetic route of 2-amino-1,3,4-oxadiazole conjugates.

Researchers used different oxidants for increasing the yield of 2-amino-1,3,4-oxadiazoles through the cyclization of acylthiosemicarbazide (Scheme 2). It was found that 1,3-dibromo-5,5-dimethylhydantoin was a very strong oxidizing agent in the presence of KI and gives maximum yields up to 97% of Compound (**16**) (5-(3-methoxyphenyl)-1,3,4-oxadiazol-2-amine). All reaction conditions are low-cost and effective, and the product is commercially produced at gram scale.


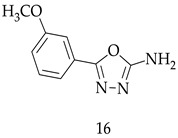


**Scheme 3:**
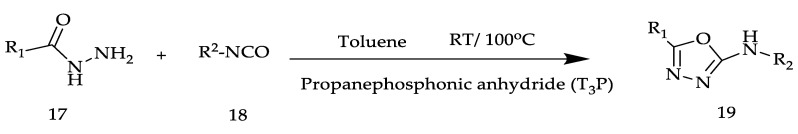
Synthetic route of 2-amino-1,3,4-oxadiazoles.

Various 2-amino-1,3,4-oxadiazoles derivatives (**19**) were synthesized by using a coupling reagent, i.e., propanephosphonic anhydride (T_3_P) from acylhydrazides (**17**) and isocyanates (**18**) by the one-pot synthesis method (Scheme 3). It is an eco-friendly method employing mild reaction condition and T_3_P preferred as a peptide coupler because of its lesser toxicity, high solubility in water, a wide range of functional group acceptability, and minimum epimerization [21].

**Scheme 4:**
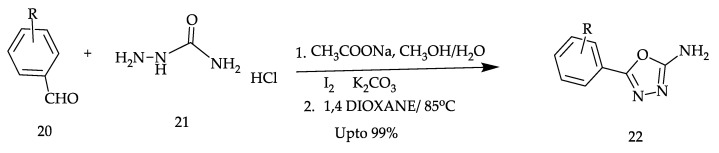
Synthetic route of 2-amino-5-substituted 1,3,4-oxadiazoles.

Pengfei Niu et al. adopted a novel synthetic route for 2,5-amino substituted 1,3,4-oxadiazoles derivatives (**22**) (Scheme 4). They used iodine as an oxidizing agent for making a C−O bond, to be utilized for ring formation by the condensation of semicarbazide (**21**) with aliphatic, as well aromatic/hetero aromatic aldehydes (**20**) followed by I_2_-mediated oxidation, which allows the synthesis of various 2-amino substituted 1,3,4-oxadiazoles derivatives with high yield [22].

**Scheme 5:**
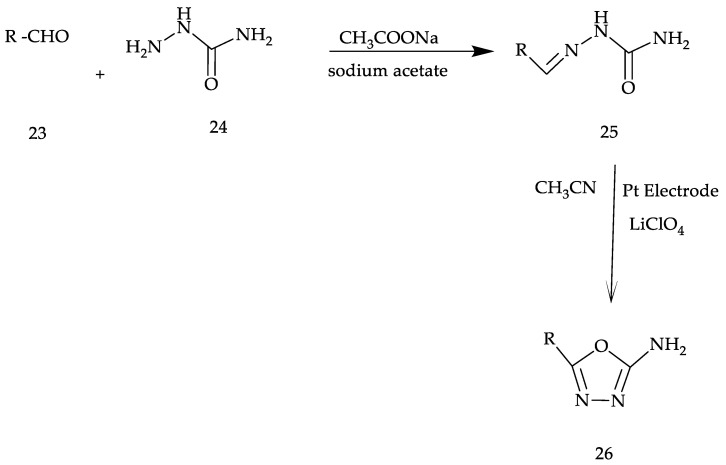
Synthetic route of 5-substituted-2-amino-1,3,4-oxadiazoles.

Another suitable electro-oxidative method was reported by Sanjeev Kumar et al. by using acetonitrile and lithium perchlorate (LiClO_4_) for the synthesis of 2-amino-5-substituted 1,3,4-oxadiazoles (**26**) at room temperature (Scheme 5). The semicarbazones (**25**) are produced as an intermediate at the platinum electrode, via reaction between substituted aldehydes (**23**) and semicarbazide (**24**) in the presence of sodium acetate [23].

**Scheme 6:**
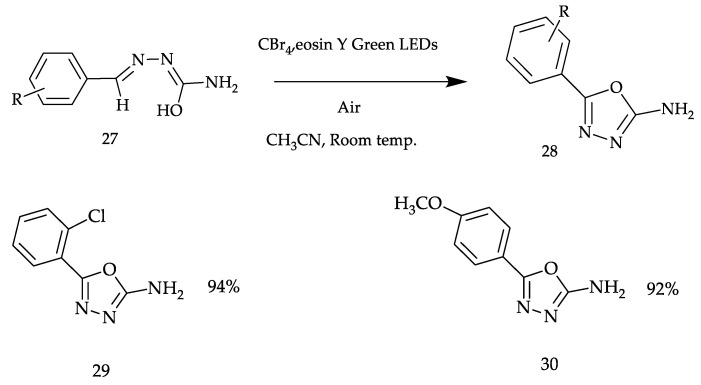
Photocatalytic oxidative approach for the preparation of 5-substituted-2-amino-1,3,4-oxadiazoles.

A proficient and high-yielding photo catalytic, a unique method for 2-amino-5-substituted-1,3,4-oxadiazoles (**28**) (Scheme 6), has been given by Kapoorr et al. Various 2-amino-1,3,4-oxadiazoles derivatives were synthesized from the oxidative heterocyclization of substituted semicarbazones (**27**) catalyzed by eosin-Y under visible-light using atmosphere oxygen andCBr4. The photo catalytic approach renders a rapid, unique, and significant method for producing substituted oxadiazoles, i.e., 5-(2-chlorophenyl)-1,3,4-oxadiazol-2-amine (**29**) and5-(4-methoxyphenyl)-1,3,4-oxadiazol-2-amine (**30**) with a yield of 94% and 92%,respectively. It is an easy and convenient method as it uses visible-light and atmospheric oxygen [24].

**Scheme 7:**
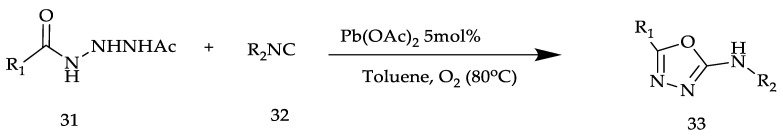
Synthetic route of 2-amino-1,3,4-oxadiazoles.

The 2-amino-1,3,4-oxadiazoles (**33**) derivatives were prepared by T. Fang et al. (Scheme 7) by using Pd-catalyzed oxidative annulations methodology, in which substituted isocyanides (**32**) reacts with substituted hydrazides (**31**) in the presence of toluene and oxygen, and undergo cyclization for producing end product [25].

**Scheme 8:**
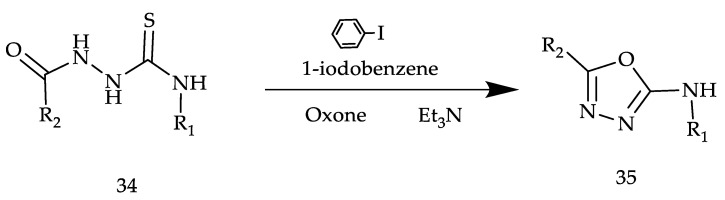
Synthetic route for 2-amino-1,3,4-oxadiazole derivatives.

Another chemical approach for the synthesis of 2-amino substituted-1,3,4-oxadiazoles given by Kavit N. Patel et al. using the hypervalent iodine reagent system. They utilized the thiosemicarbazides (**34**) oxidative desulfurization approach for the building blocks of 2-amino-1,3,4-oxadiazole compounds (**35**) (Scheme 8) using both reactants, i.e., first iodobenzene, then Oxone. The chemical properties of catalysts provide versatility for desulfurization reaction [26].

**Scheme 9:**
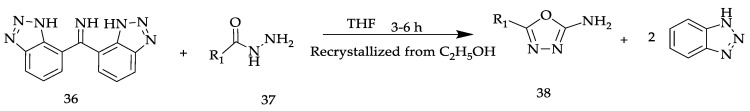
Synthetic route of 5-Aryl-2-amino-1,3,4-oxadiazole derivatives. R_1_ = Ph, 4-t-BuC_6_H_4_, 4-NH_2_ C_6_H_4_, 4-OH C_6_H_4_, 4-NO_2_ C_6_H_4_, 3-NO_2_ C_6_H_4_, 4-Cl C_6_H_4_, 2-Cl C_6_H_4_, 4-Pyridyl, EtO.

Katritzky et al. synthesized 5-aryl-2-amino-1,3,4-oxadiazole compounds (**38**) (Scheme 9) in high yields via a reaction between Di(benzotriazolyl) methanimine (**36**) and substituted arylhydrazides (**37**), being dissolved in dry THF andrefluxed for 3–6 h [27].

**Scheme 10:**
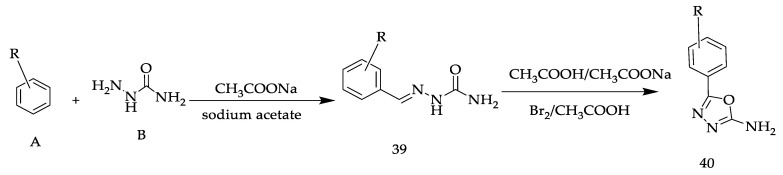
Synthesis of 2-amino-5-aryl-1,3,4-oxadiazoles.

H. Rajak et al. synthesized 5-substituted-2-amino-1,3,4-oxadiazole derivatives by using oxidative cyclization of semicarbazones (**39**) (Scheme 10), in the presence of bromine, in acetic acid in alkaline medium. Substituted semicarbazone derivatives were prepared by reacting substituted aldehydes (A) with semicarbazide (B) in the presence of sodium acetate [28].

**Scheme 11:**
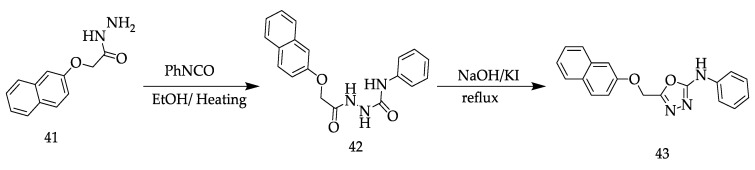
Synthesis of 5-((naphthalen-2-yloxy) methyl)-N-phenyl-1,3,4-oxadiazol-2-amine.

Another proficient approach for the synthesis of new 2,5-disubstituted-1,3,4-oxadiazole derivatives was reported by El-Sayed et al. They synthesized 5-((naphthalen-2-yloxy) methyl)-N-phenyl-1,3,4-oxadiazol-2-amine (**43**) by the cyclization reaction of acylthiosemicarbazides (**42**), in the presence of NaOH, using KI as the oxidizing agent (Scheme 11) followed by the heating of 2-[(naphthalen-2-yloxy)acetyl]-N-phenylhydrazine carboxamide (**41**) with ethanol [29].

**Scheme 12:**
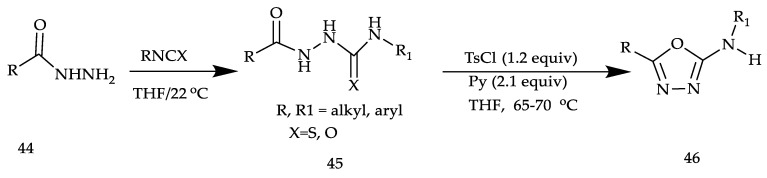
Synthetic route for 5-aryl-2-amino-1,3,4-oxadiazole derivatives.

Dolman J. Sarah et al. reported the synthesis of various 5-aryl-2-amino-1,3,4-oxadiazoles (**46**) using a unique method, which involves the cyclization of semi carbazides (**45**) (prepared by the acylation of a given hydrazide (**44**) with an appropriate isocyanate (Scheme 12). In the presence of tosyl chloride/pyridine, this method produces a wide variety of oxadiazoles [30].

**Scheme 13:**
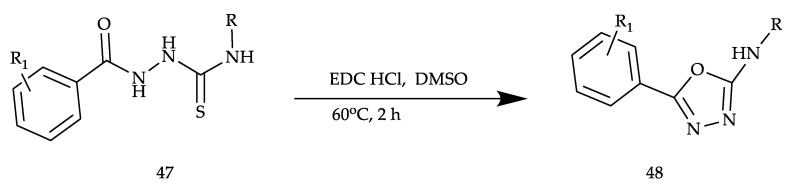
Synthesis of 2amino-substituted 1,3,4-oxadiazole.

Another convenient EDC·HCl-, i.e.,the regioselective reagent-based cyclization method, was reported by Yang, S. J etal. They synthesized the synthesis of 2-amino-substituted 1,3,4-oxadiazole (**48**) from thiosemicarbazide (**47**). The EDC·HCl (1-ethyl-3-(3-dimethylaminopropyl) carbodiimide is a effective desulfurizing agent. The main advantage of this method was regioselectivity and high yields (Scheme 13) [31].

### 2.1. Various Synthesis Methods for 5-Substituted-1,3,4-Oxadiazole-2-Thiols

**Scheme 14:**
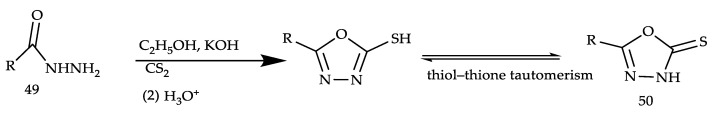
Synthesis of 5-substituted-1,3,4-oxadiazole-2-thiols.

The unique route for the synthesis of 5-substituted-1,3,4-oxadiazole-2-thiol(thione)s (**50**) (Scheme 14) was reported by Koparir M. et al., which involves a reaction between an acylhydrazides (**49**) and CS2 in an alcoholic alkaline solution, followed by the acidification of the reaction mixture. A large number of 1,3,4-oxadiazole derivatives have been reported in recent years by this core route. The tautomerism phenomenon also exist for thiol-thione (**50**) in the synthesized compounds [32].

### 2.2. Various Synthesis Methods for 2,5-Diaryl(alkyl/thiol)-1,3,4-Oxadiazole

Various synthetic routes for 2,5-Diaryl(alkyl/thiol)-1,3,4-oxadiazole have been reported in the literature using dehydrating agents such as phosphorous oxychloride (POCl_3_), phosphorus pentachloride, sulfuric acid, phosphorus pentoxide, phosphoric acid, thionyl chloride, trifluoroacetic acid, and milder reagents such as carbodiimide derivatives, etc. [33,34].

**Scheme 15:**
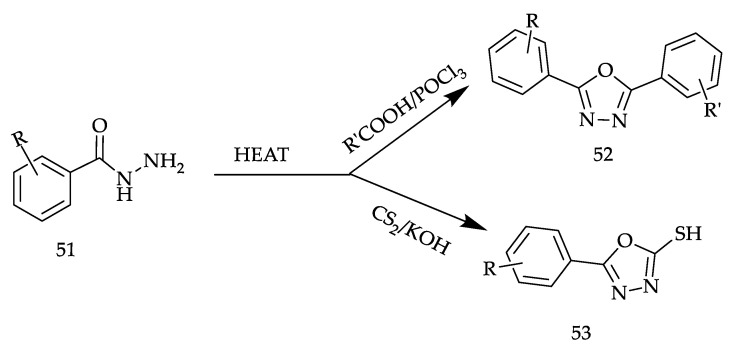
Synthesis of 2,5-disubstituted-1,3,4-oxadiazoles.

Keshari K. Jha et al. reported a very simple and easy method of preparation for 1,3,4-oxadiazole (**52**–**53**) (Scheme 15) derivatives by the ring closure reactions of various acylhydrazides (**51**) with carbon disulphide in alkaline solution, and with aromatic acids in POCl3. This method produced a final product in high yield. Arylhydrazides (**51**) were synthesized from various aromatic acids in the presence of H_2_SO_4_ as a catalyst, via esterification [35].

**Scheme 16:**
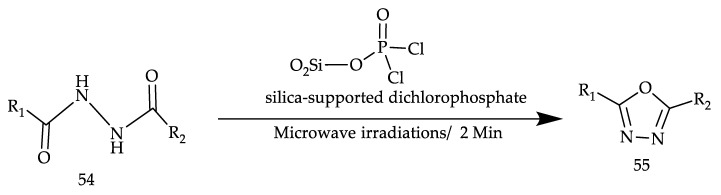
Synthesis of 2,5-disubstituted 1,3,4-oxadiazoles.

Using an efficient and suitable cyclodehydration agent, i.e.,dichlorophosphate (silica-supported), for the synthesis of 2,5-disubstituted 1,3,4-oxadiazoles (**55**) from 1,2-diacylhydrazines (**54**) by using microwave irradiation in solvent-free medium, was reported by Zhu, Li. et al. [36] (Scheme 16). A major advantage of the method is that it is suitable for alkyl, aryl, and heterocyclic-substituted 1,3,4-oxadiazoles, and is free from corrosion or environmental pollution.

**Scheme 17:**
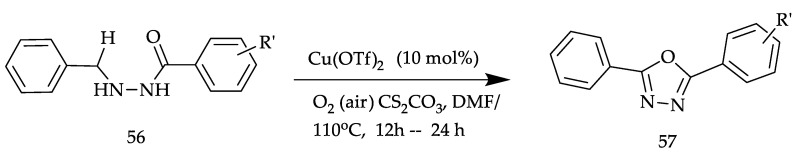
Synthesis of 2,5substituted diaryl 1,3,4-oxadiazoles.

Another convenient route for the synthesis of symmetrical and unsymmetrical 2,5-disubstituted-1,3,4-oxadiazoles (**57**) was reported by Guin et al. by using Cu(OTf)2 as a catalyst for the imine C-H functionalization of N-arylidenearoylhydrazide (**56**) (Scheme 17) [37].

**Scheme 18:**
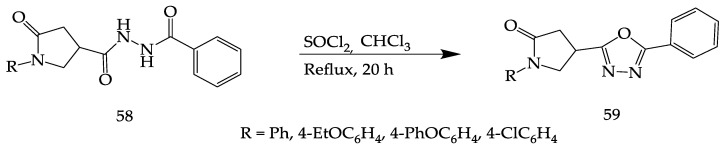
Synthesis of 1-aryl-4-(5-aryl-1,3,4-oxadiazol-2-yl)pyrrolidin-2-ones.

Mickeviciuset al. synthesized 2,5-disubstituted-1,3,4-oxadiazoles by the dehydration of acylhydrazides by using a dehydrating agent (thionyl chloride). They were synthesized by various 1-aryl-4-(5-aryl-1,3,4-oxadiazol-2-yl)pyrrolidin-2-one (**59**) derivatives from different acid hydrazides (**58**), using thionyl chloride in high yields (Scheme 18) [38].

**Scheme 19:**
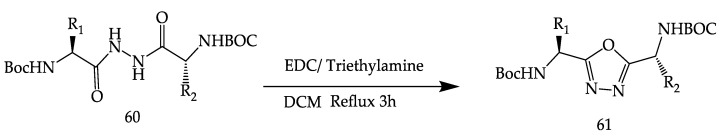
Synthesis of orthogonally protected 1,3,4-oxadiazole derivatives.

Another high yielding, efficient, and suitable method was reported by Nagendra et al. for the synthesis of orthogonally protected 1,3,4-oxadiazole derivatives (**61**) (70–92%) by the cyclodehydration of diacylhydrazines using 1-ethyl-3-(3-dimethylaminopropyl) carbodiimide (EDC) as a dehydration agent (Scheme 19) [39].

## 3. Evidence of the Biological Potential of 1,3,4-Oxadiazole Derivatives

**Figure 7:**
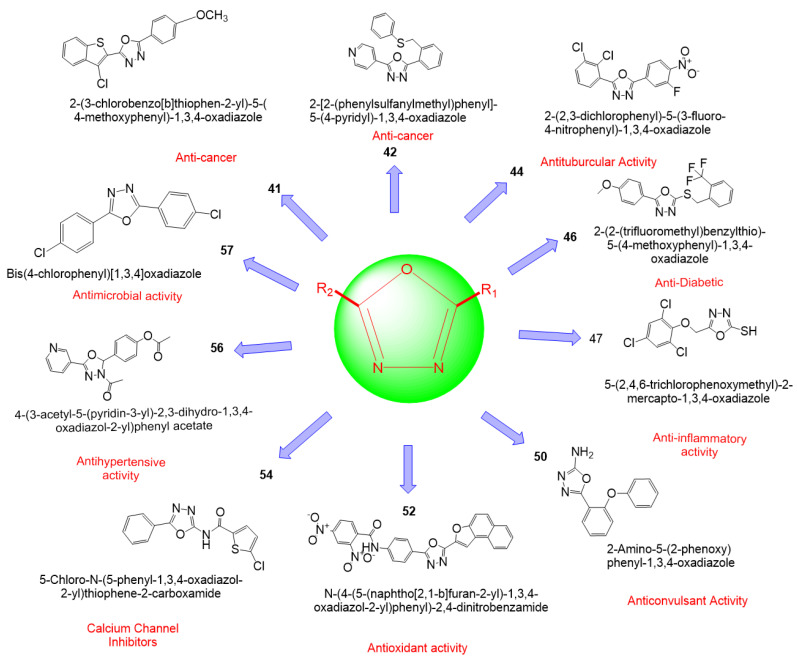
The biological activity of 1,3,4-oxadiazole derivatives.

The 1,3,4-oxadiazole scaffold exhibits a high bioactivity and the specificity of binding; therefore, it could be an attractive option for researchers. A broad spectrum of biological activities are exhibited by 1,3,4-oxadiazole derivatives, which are used as leading compounds for developing chemical drugs. We present an overview of 1,3,4-oxadiazole derivatives that, during the past decade, have been synthesized and screened for their antimicrobial, antitumor, and antiviral antioxidants, and many more activities (Figure 7). The purpose of this review was to discuss the synthetic development of 1,3,4-oxadiazole derivatives, as well as their bioactivity and potential as cytotoxic agents. Therefore, a moiety of 1,3,4-oxiadiazole has been reported to be effective as being anticancer [40,41,42], antitubercular [43,44], antidiabetic [45], anti-inflammatory [46,47,48], anticonvulsant [49,50], andantioxidant [51,52], in addition to being calcium channel inhibitors [53,54], having antihypertensive activities [55], being antimicrobial [56,57], a pesticide [58,59], being MAO inhibitors [60], tyrosinase inhibitors [61], and cathepsin K inhibitors [62], etc.

## 4. Biochemical Mechanisms Leading to Cancer

The uncontrolled division of cancer cells is a hallmark of this deadly disease. In the current era, chemotherapy is the most common method of cancer treatment, either alone or in combination with surgery and radiotherapy. The demand for new cancer therapies is increasing as researchers and clinicians consider this solution to the drug shortage dilemma. Modern genomic and proteomic technologies have steadily increased the number of proposals for new cancer drug targets, based on the assessment of cancer-specific biological pathways. Many proteins and enzymes such as cyclin-dependent kinases (CDKs) participate in the regulation of cell proliferation and the phase of the cell cycle-G0/G1, S, G2, and M. Consequently, the regulation of cells and their phases is regarded as a promising target for the development of new anticancer molecules. Therefore, targeted therapies require an understanding of how cell-cycle and their regulations, genes regulations, and biochemical pathways affect cancer behavior. In this challenging disease, molecularly specific therapies have provided some of the most significant advances, including monoclonal antibodies and small-molecule tyrosine kinase inhibitors. The findings of this study will demonstrate that cell proteins, growth factors, and numerous biological pathways could be selective targets to develop new anticancer drugs. The review also emphasizes the various anticancer properties of 1,3,4-oxadiazole scaffolds, which target primarily enzymes and kinases. Many targets exist, but few are here, namely:

1. Telomerase enzyme;
2. Histone deacetylase (HDAC);
3. Thymidylate synthase;
4. Thymidine phosphorylase enzyme;
5. Other evidence of the anticancer properties of 1,3,4-oxadiazole derivatives.

### 4.1. Telomerase

**Figure 8:**
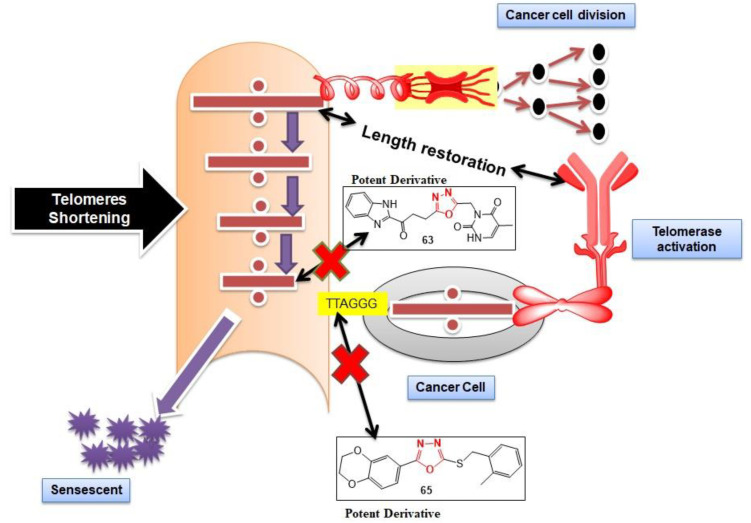
Telomerase enzyme and its inhibitors.

Telomerase is specialized ribonucleoprotein that is found in mammalian cells, having a very specific function for the maintenance and stability of telomere, for the functionalization of chromosomal integration and cell proliferation [63,64,65]. In most somatic cells, during the DNA replication process, the telomere length decreases (known as the mitotic clock). When the telomere’s length became too short, the metabolism becomes slower, causing genomic instability, thereby stopping the proliferation of cells. This phenomenon is called senescence. However, this ribonucleoprotein becomes activated in cancerous cells by adding extra nucleotide sequence, TTAGGG, and becomes stabilized (Figure 8).

**Figure 9:**
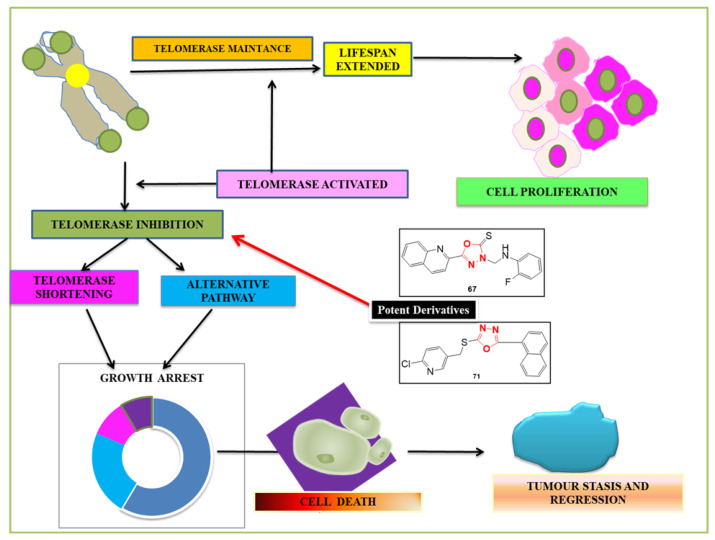
Telomerase enzyme and cell proliferation.

So, tumor progression may be prevented by inhibiting the addition of extra nucleotide sequence, TTAGGG, or by inhibiting the shortening of the telomere. Therefore, telomere may be considered a potential target for the development of telomerase inhibitors in carcinogenesis, due to its role in continuous cell division and preventing replicative senescence [66,67] (Figure 9).

#### 4.1.1. The Role of Telomerase Enzyme in Cells

**Figure 10:**
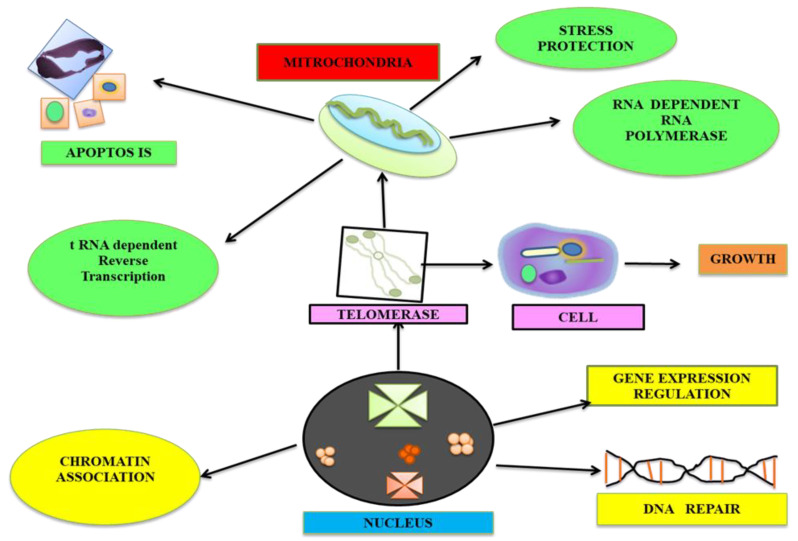
Various functions of the telomerase enzyme.

The telomerase enzyme plays a significant role in telomere lengthening, mitochondria, and nucleus protection. Inside the cell, the telomerase enzyme improves cell growth by: decreasing apoptosis [68,69]; decreasing stress causing factors [70];repairing DNA [71,72]; contributing to gene expression and its regulations [73,74,75]; RNA polymerization and reverse transcriptions [76]; chromatin activation for replication [77,78]; cell survival factors; and promoting neuron-protective signaling [79], as shown in Figure 10.

#### 4.1.2. Telomeres Association Proteins

**Figure 11:**
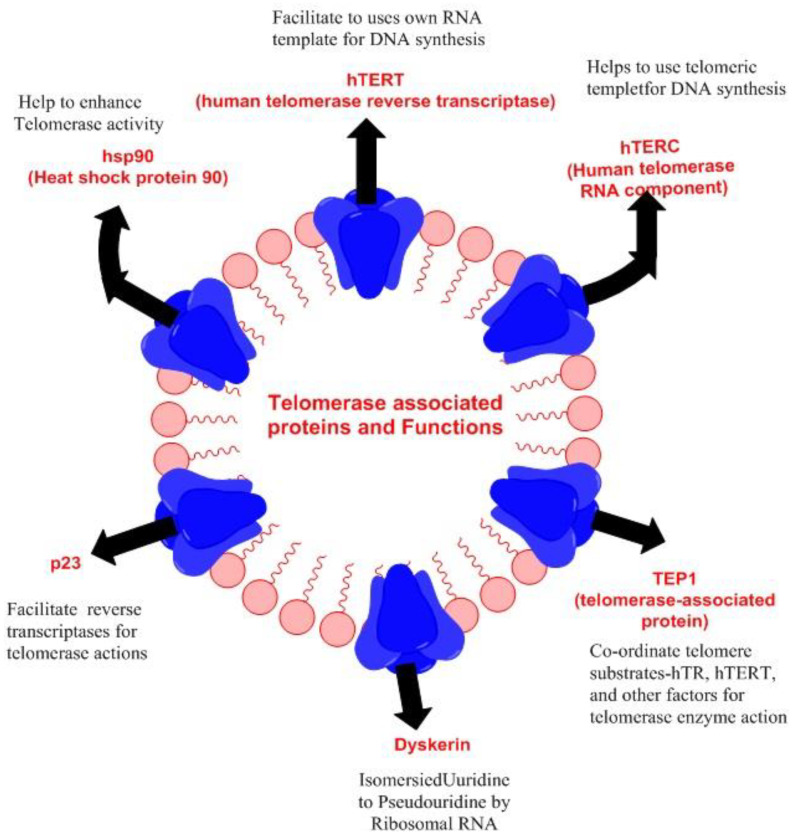
Telomerase proteins and their functions.

Telomeres protect exonucleolytic degradation and the fusion of chromosome by forming helical loop structures around chromosomes. The telomerase enzyme regulates telomere lengthening for chromosomal integrity via various chemical reactions in DNA, including reverse transcription and translocation [80]. Chromosomal integrity is regulated by telomerase via the other six protein subunits which are associated with it. The six proteins subunits of telomerase, i.e., hTERC, hsp90, hTERT, TEP1, p23, and dyskerin, together with their functions, are summarized in Figure 11 [80,81]. Telomerase activity is primarily regulated by human telomerase RNA components, and human telomerase reverse transcriptase subunits [82].

#### 4.1.3. Telomerase Inhibitors

**Figure 12:**
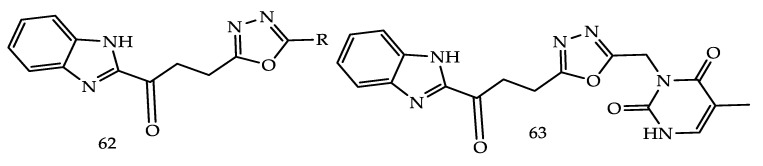
Chemical structures of benzimidazole moiety containing 1,3,4-oxadiazole.

Asif Husain et al. synthesized a benzimidazole moiety containing 1,3,4-oxadiazole library (**62**) (Figure 12) from 4-(1H-benzo[d]imidazol-2-yl)-4-oxobutanehydrazide, and screened all the derivatives against CCRF-CEM (leukemia), MDA-MB-435 (melanoma), MLT-4 (leukemia), CCRF-CEM (leukemia) and K-562 (leukemia) cancer cell lines. Among the selected 19 derivatives, Compound (**63**) (Figure 12) has shown a significant amount of cytotoxicity potential against the cell lines, i.e., the MID GI50 value of 2.09 (mean graph midpoint (arithmetical mean value of treated cancer cell lines)) was observed, which was significant value as a comparison to marketed anticancer drugs, i.e., bendamustine with MID GI50 value 60, and chlorambucil with GI50 value 52, respectively [83].

**Figure 13:**
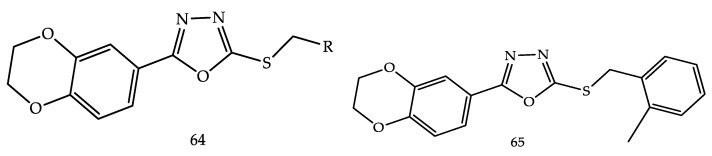
Chemical structures of 1,4-benzodioxan moiety containing 1,3,4-oxadiazole derivatives.

A new series of 1,4-benzodioxan moiety containing 1,3,4-oxadiazole derivatives (**64**) (Figure 13) was prepared by Zhang et al. and screened for telomerase inhibitory activity by TRAP-PCR-ELISA assay [84].

The synthesized derivatives were tested against four different cancer cell lines, HEPG2, HELA, SW1116, and BGC823, compared with positive control 5-fluorouracil, a well-known anticancer agent. Among all derivatives, Compound 2-(2,3-Dihydrobenzo[b][1,4]dioxin-6-yl)-5-(2-methylbenzylthio)-1,3,4-oxadiazole (**65**) was found to possess the most potent telomerase inhibitory action (IC_50_ = 1.27 ± 0.05 µM).

**Figure 14:**
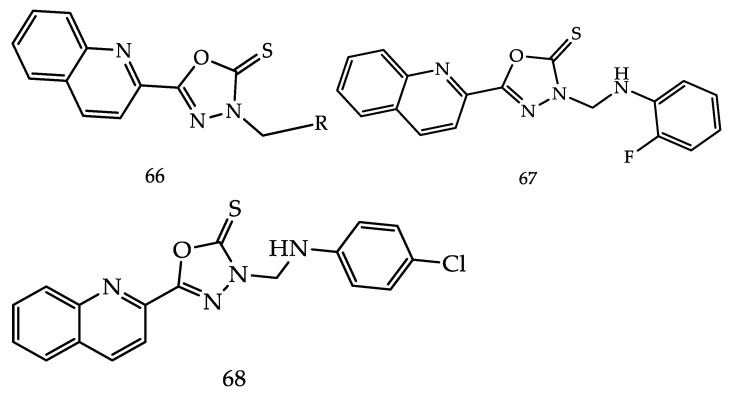
The 2-aminomethyl-5-(quinolin-2-yl)-1,3,4-oxadiazole-2(3H)-thione quinolone derivatives.

A novel series of 2-aminomethyl-5-(quinolin-2-yl)-1,3,4-oxadiazole-2(3H)-thione quinolone derivatives (**66**) was synthesized by Sun et al. (Figure 14). Telomerase inhibitors were synthesized and tested against HepG2 (human hepatoma cells), SGC-7901 (human gastric cancer cells), and MCF-7 (human breast cancer cells). Telomerase inhibitory effects were demonstrated for most of the compounds tested. Among them, compounds 3-(((2-Fluorophenyl) amino) methyl)-5-(quinolin-2-yl)-1,3,4-oxadiazole-2(3H)-thione (**67**) and 3-(((4-Chlorophenyl) amino) methyl)-5-(quinolin-2-yl)-1,3,4-oxadiazole-2(3H)-thione (**68**) showed the maximum antitumor effect via telomerase inhibition with IC_50_ 0.8 ± 0.1 and 0.9 ± 0.0 mM [85].

**Figure 15:**
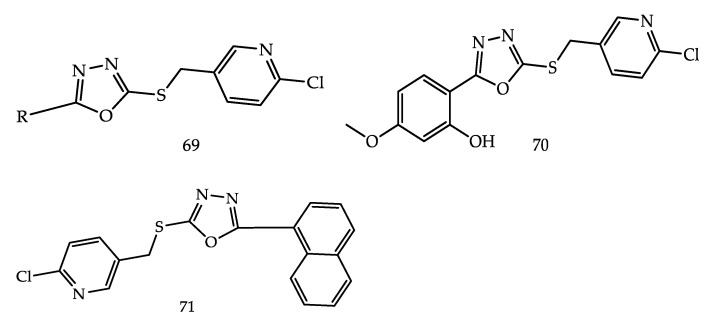
The chemical structure of 2-chloropyridine derivatives possessing 1,3,4-oxadiazole moiety.

Zheng et al. synthesized novel 2-chloropyridine derivatives possessing 1,3,4-oxadiazole moiety (**69**) (Figure 15) and evaluated telomerase inhibitory activity against gastric cancer cell lines SGC-7901 by modified TRAP (telomere repeat amplification protocol). Among all derivatives, Compound (**70**) and (**71**) showed significant telomerase inhibitory activity (IC_50_ = 2.3 ± 0.07 µM and 2.56 ± 0.11, respectively) as compared to the positive control ethidium bromide (IC_50_ = 2.5 ± 0.23). A docking binding model and structure activity relationship showed that electron-donating groups on the ortho position of the benzene ring had lower inhibitory activity than those on the para position [86].

**Figure 16:**
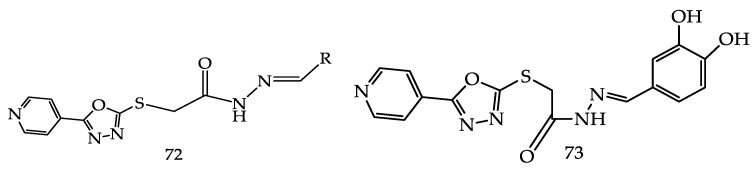
The chemical structure of pyridine and acylhydrazone bearing 1,3,4-oxadiazole moiety.

A novel series of potent 1,3,4-oxadiazole derivatives bearing pyridine and acylhydrazonemoieties (**72**) (Figure 16) were synthesized by Zhang et al. and were screened by TRAP PCR-ELISA assay for anticancer activity. Compound (E)-N’-(3,4-dihydroxybenzylidene)-2-((5-(pyridin-4-yl)-1,3,4-oxadiazol-2-yl)thio)acetohydrazide (**73**) was found to be the most potent inhibitor against four cancer cell lines (HEPG2, MCF7, SW1116, BGC823) with an IC_50_ value of 1.18 ± 0.14 µM, which is lower than that of the positive control staurosporine (4.18 ± 0.05 µM) and ethidium bromide(2.71 ± 0.18 µM) [87].

## 5. Histone Deacetylase (HDAC) Functions and Its Inhibitors

**Figure 17:**
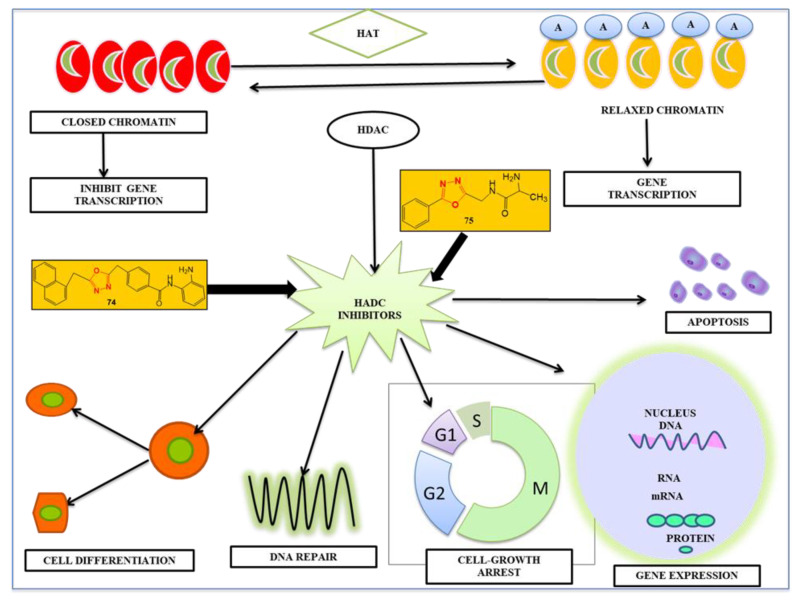
Histone deacetylase (HDAC) and its functions.

Human histone deacetylases (HDACs) comprise 18 proteins that span four different protein classes. In carcinogenesis, these proteins play a role in transcription, gene regulation, mutation, and protein encoding. Therefore, HDAC inhibitors, are relatively new classes of anticancer agents that induce cell death, cell apoptosis, and seize cancer cell cycle. They play significant roles in gene regulation, cell death, and apoptosis in cancer cells [88,89]. Histone as a protein performs a very important role for acetylation and deacetylation by HDACs and HATs (histone acetyl-transferases), which eliminate acetyl groups from DNA-binding histone proteins, thereby lowering the uses of chromatin for transcription factors, and preventing transcription inside genetic material which regulate cell proliferation and cell death (Figure 17) [90].

Valente et al. synthesized a new series of hydroxamates, or 2-aminoanilides containing 1,3,4-oxadiazole derivatives, and studied histone deacetylase enzyme inhibition. Anti-proliferative studies conducted against colon carcinoma (SW620) and myeloid leukemia (HL60, HEL, KG1) cell lines showed HDAC inhibition, which was correlated with the induction of cell growth arrest, apoptosis, and cell differentiation [91].

**Figure 18:**
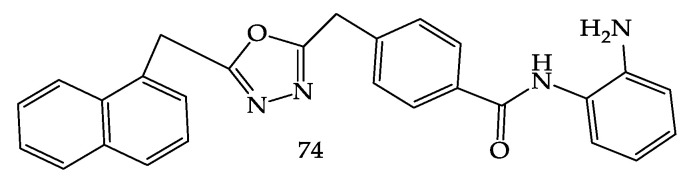
N-(2-Aminophenyl)-4-((5-(naphthalen-1-ylmethyl)-1,3,4-oxadiazol-2-yl)-methyl) benzamideas HDAC inhibitor.

Among all derivatives, Compound N-(2-Aminophenyl)-4-((5-(naphthalen-1-ylmethyl)-1,3,4-oxadiazol-2-yl)-methyl)benzamide (**74**) (Figure 18) was found to be the most potent and selective inhibitor against HDAC, against vorinostat (Histone deacetylase inhibitor) as a reference drug.

**Figure 19:**
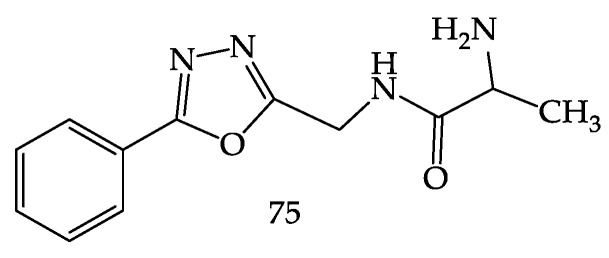
The 2-amino-N-((5-phenyl-1,3,4-oxadiazol-2-yl) methyl) propanamide as HDAC inhibitor.

Various 1,3,4-oxadiazole derivatives were composed of different amino acids, i.e., glycine or alanine, and evaluated against HDAC-8, enzyme [92]. The Compound 2-amino-N-((5-phenyl-1,3,4-oxadiazol-2-yl) methyl) propenamide (**75**) (Figure 19) was found to be a prominent derivative with the highest inhibitory effect against breast cancer cell lines growth (MCF-7 and MDA-MB-231),via in vitro cell lines studies [93].

## 6. Thymidylate Synthase and Its Inhibitors

**Figure 20:**
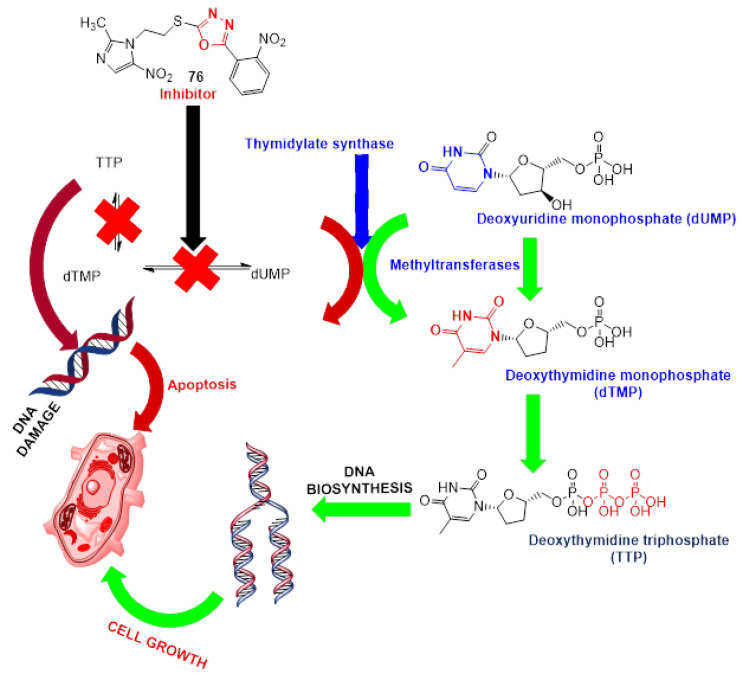
Thymidylate synthase and its inhibitors.

The DNA replication process is a very complex process and many enzymes are involved in its biosynthesis, transcription, and repair. Among all enzymes, thymidylate synthase is an essential enzyme that shows a significant contribution in its replication. Further, TS helps in the conversion of dUMP to dTMP (Figure 20) for producing thymine via thymidylic acid, which is the building block of nucleic acid, an essential unit of DNA. This inactivation of TMP leads to a decrease in the dTTP, causing disturbance in the DNA biosynthesis, and slows down the growth and proliferation of the cell [94].

The molecule targeting towards TS inhibition via similar analogues of thymidylate synthase substrates creates an imbalance in the conversion of the dUMP to dTMP pool. Further, DNA synthesis in cancer cells is higher than in normal cells. This chemical mechanism (the diminution of thymidylic acid in the cells via TS inhibition) may attract researchers towards the development of new anticancer drugs. Many TS inhibitors are used in chemotherapy for the treatment of ovarian, gastric, breast, and colorectal cancers [95].

**Figure 21:**
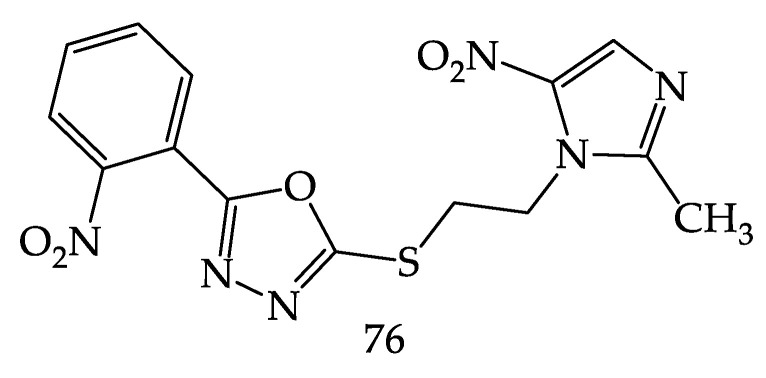
The 2-((2-(2-methyl-5-nitro-1H-imidazol-1-yl)ethyl)thio)-5-(2-nitrophenyl)-1,3,4-oxadiazoleas thymidylate synthase inhibitor.

Qian-Ru Du et al. prepared newer thioether composed of 1,3,4-oxadiazole derivatives and tested as thymidylate synthase enzyme inhibitor for anticancer activity. During the research study, they found 2-((2-(2-methyl-5-nitro-1H-imidazol-1-yl) ethyl)thio)-5-(2-nitrophenyl)-1,3,4-oxadiazole (**76**) (Figure 21) was the most potent against breast cancer (MCF-7), stomach cancer (SGC-7901), and liver cancer lines (HepG2) (IC_50_ values of 0.7 ± 0.2, 30.0 ± 1.2, 18.3 ± 1.4 μM), respectively, in comparison to 5-fluorouracil (IC_50_ values 22.8 ± 1.2, 28.9 ± 2.2 and 16.7 ± 1.5) as a standard anticancer drug. Compound (**76**) exhibited high inhibitory potential against human thymidylate synthase (IC_50_ value—0.62 μM) as compared to raltitrexed as a positive control drug. This effect is 10 times more potent for bacterial thymidylate synthase inhibitory effect (isolated from *E. coli*) (IC_50_ value 0.47 μM) [96].

**Figure 22:**
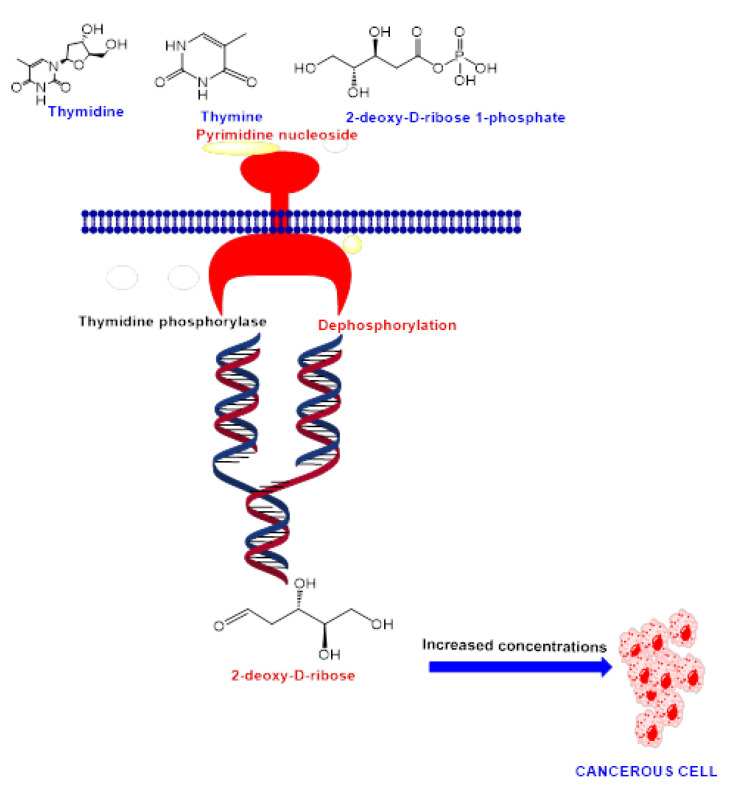
Thymidine phosphorylase enzyme.

Platelet-derived endothelial cell growth factor is also known as thymidine phosphorylase (TP). This enzyme is responsible for the reversible conversion of thymidine to thymine (pyrimidine nucleoside degraded) inside the nucleic acid, and causes proliferation. Thymidine, thymine, and 2-deoxy-D-ribose 1-phosphate together form pyrimidine nucleoside, which gives 2-deoxy-D-ribose after dephosphorylation (Figure 22). This is identified via in vitro as well as in vivo studies by the stimulation of chemotactic and VEGF (vascular endothelial growth factor) secretion for angiogenic activity [97,98].

Therefore, a higher concentration of thymidine phosphorylase inside cell signaling may lead to uncontrolled cell division, or cancerous cells. Thymidine phosphorylase enzyme may also become activated by physical and chemical stress in the cancer tissue, that promotes the concentration of 2-deoxy-D-ribose, which is the causative factor for the progression of tumors. So, the platelet-derived endothelial cell growth factor becomes a very important target for the development of novel thymidine phosphorylase inhibitors as anticancer drugs [99,100].

Khan et al. prepared various 2,5-substituted 1.3.4-oxadiazoles and evaluated all molecules against thymidine phosphorylase activity. Among all of the tested derivatives, Compound 2,5-di(pyridin-3-yl)-1,3,4-oxadiazole (**77**) was the most potent as an enzyme inhibitor having 3-pyridyl substituent at positions 2 and 5, against 7-deazaxanthin taken as a standard drug [101].


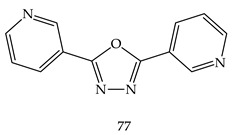


Ullah and Javid et al. made indoline and hydrazone clubbed 1,3,4-oxadiazole derivatives and checked for their thymidine phosphorylase inhibition activity by comparing them with 7-deazaxanthin as a standard drug. The most potential compounds were (Z)-N’-(4-hydroxy-3,5-dimethoxybenzylidene)-4-(5-(4-isocyanophenyl)-1,3,4-oxadiazol-2-yl)benzohydrazide (**78**) (hydrazone derivatives) and(Z)-1-((5-(3-hydroxy-4-methoxyphenyl)-1,3,4-oxadiazol-2-yl)imino)-3-isopropyl-1,3-dihydro-2H-inden-2-one (**79**) (from the indoline series), which had 30 and 08 times more phosphorylase inhibition potentialincomparison to a standard drug, respectively [102].


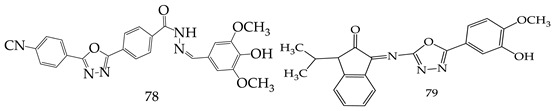


The 5-(4-chlorophenyl)-1,3,4-oxadiazole-2-thione derivatives were synthesized by Bajaj et al. and evaluated their anticancer potential (on the breast cancer cell line (MCF-7) for thymidine phosphorylase inhibition activity. Researchers found derivatives (**80**) and (**81**) with the highest potency against breast cancer cell line (MCF-7), as well as thymidine phosphorylase enzyme inhibitory activity, as compared to the reference drugs adriamycin and 7-deazaxanthin, respectively [103].


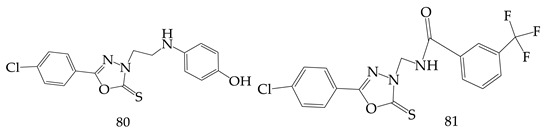


Taha M et al. synthesized novel derivatives of (bis-5-chloro-indol-3-yl) methyl clubbed 1,3,4-oxadiazoles and evaluated them against thymidine phosphorylase enzyme inhibitory activity. The most potent derivatives were 4-(5-(4-(bis(5-chloro-1H-indol-3-yl)methyl)phenyl)-1,3,4-oxadiazol-2-yl)benzene-1,3-diol (**82**), 3-(5-(4-(bis(5-chloro-1H-indol-3-yl)methyl)phenyl)-1,3,4-oxadiazol-2-yl)benzene-1,2-diol (**83**), 2-(5-(4-(bis(5-chloro-1H-indol-3-yl)methyl)phenyl)-1,3,4-oxadiazol-2-yl)benzene-1,4-diol (**84**), which differed with the different position of hydroxyl groups in the phenyl ring. They had6–9 times more thymidine phosphorylase inhibitory action as compared to 7-deazaxanthin as a reference drug [104].


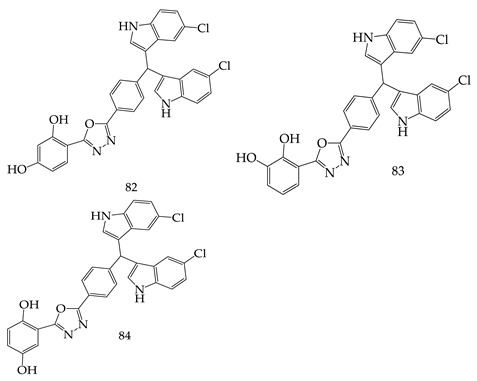


## 7. Miscellaneous Anticancer Properties of 1,3,4-Oxadiazole Derivatives

Salahuddin et al. synthesized various substituted-(1,3,4-oxadiazol-2-yl) quinoline derivatives (**85**,**86**) and screened out a whole prepared series for anticancer activity from the National Cancer Institute (USA) at the concentration of single dose (10^−5^ M). Among all the tested compounds, 2-(2-chloroquinolin-3-yl)-5-(3,5-dimethoxyphenyl)-1,3,4-oxadiazole (**87**) showed extended potency with 95.70% growth (mean growth percent %) against SNB-75 (CNS cancer) and UO-31 (renal cancer) (growth % of the most sensitive cell line 53.35 and 64.35). The Compound 2-(2-chloroquinolin-3-yl)-5-((2-(phenoxymethyl)-1H-benzo[d]imidazol-1-yl)methyl)-1,3,4-oxadiazole (**88**) showed 96.86 GP and was highly active on SNB-75 (CNS cancer GP 51.27) [105].


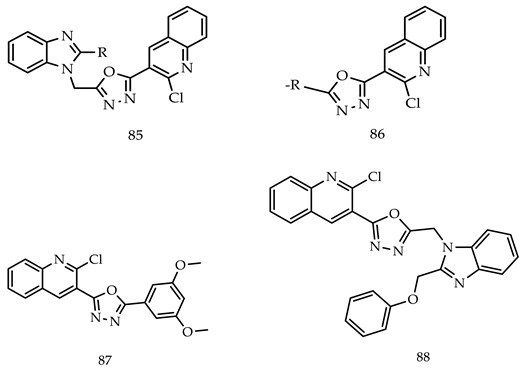


Shamsuzzaman et al. synthesized new anticancer steroidal oxadiazole, pyrrole and pyrazole derivatives, and tested them against human leukemia cell line HL-60 by MTT assay. Their findings showed that 3′-[5′-mercapto-1,3,4-oxadiazole-2-yl] methoxy cholest-5-en (**89**) was the most powerful anticancer compound with an IC_50_(17.33) value [106].


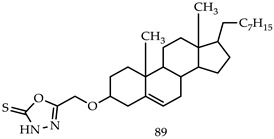


B.S Holla et al. synthesized various 2-chloro-1,4-bis(5,2-substituted-1,3,4-oxadiazol-2-ylmethyleneoxy) phenylene derivatives, which were synthesized and tested for anticancer activity against different types of human cancer cells lines (breast, ovarian, colon, renal prostrate, lung, etc.,). Among the all derivatives, Compounds 5,5’-(((2-chloro-1,4-phenylene)bis(oxy))bis(methylene))bis(2-((2,4-dichlorophenoxy)methyl)-1,3,4-oxadiazole) (**90**) and 5,5’-(((2-chloro-1,4-phenylene)bis(oxy))bis(methylene))bis(2-((4-chlorophenoxy)methyl)-1,3,4-oxadiazole) (**91**) showed significant anticancer potential against all the cancer cell lines with GI50 values at less than 10 µM. Molecule (**90**) demonstrated the highest anticancer activity against all cell lines, i.e., HL-60 (leukemia), RPMI-8226(leukemia), SR (leukemia), and UACC-62 (melanoma), with GI50 values 3.52, 9.51, 0.03, and 4.65 µM, respectively [107].


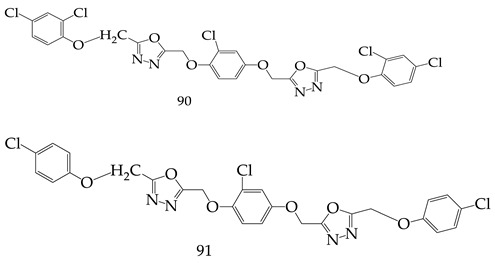


More than 50 cancer cell lines were tested forin vitro anticancer activity using dihydropyrimidine (DHPM) derivatives bearing an 1,3,4-oxadiazole moiety as monastrol analogues at the National Cancer Institute, USA, by F. A. Ragab et al [108]. The most potent derivatives against leukemia HL-60(TB) cell line were (ethyl 4-(3-chlorophenyl)-2-(((5-(4-chlorophenyl)-1,3,4-oxadiazol-2-yl)methyl)thio)-6-methyl-1,4-dihydropyrimidine-5-carboxylate) (**92**) and (ethyl 2-(((5-(4-chlorophenyl)-1,3,4-oxadiazol-2-yl)methyl)thio)-4-(2,4-dichlorophenyl)-6-methyl-1,4-dihydropyrimidine-5-carboxylate) (**93**) with IC_50_ = 0.056 µM and 0.080 µM, respectively, being more active than monastrol (IC_50_ = 0.086 µM).


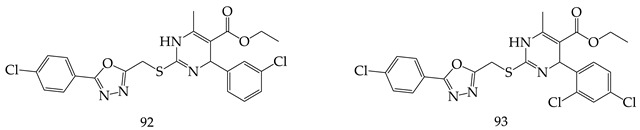


Abdel K. Mansour et al. synthesized new complex 1,3,4-oxadiazole derivatives from carbohydrazides. Among the tested derivatives 2-(1,3,4-triphenylpyrazol-5-yl)-5-phenyl-1,3,4-oxadiazole (**94**) and 2-(4-phenyl-5-benzoylamino-1,3-diphenyl-2-pyrazolin-5-yl)-1,3,4-oxadiazol-5(4H)-thione (**95**) were found with in vitro anticancer potency, and Compound (**94**) showed most promising (concentration 10^−4^ M) activity against leukemia cell lines (CCRF-CEM, K-562, MOLT-4, PRMI-8226, SR) [109].


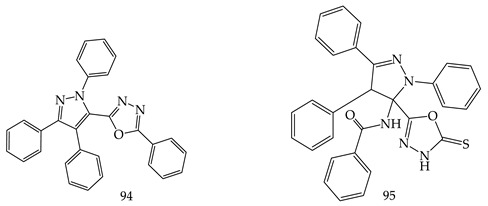


Another new series of 1,3,4-oxadiazole derivatives possessing sulfonamide moiety (**96**) were designed and synthesized by El-Din et al. and screened for in vitro antiproliferative activities against NCI-58 human cancer cell lines of nine different cancer types. Out of them, Compound [N-(4-((5-(4-Chlorophenyl)-1,3,4oxadiazol-2-yl) methoxy)-3-fluorophenyl)-4 methoxybenzenesulfonamide] (**97**) exerted high potency, efficacy, and broad-spectrum antiproliferative activity. The % inhibition values of Compound (**97**) over T-47 D breast, SR leukemia, SK-MEL-5 melanoma, and MDA-MB-468 breast cancer cell lines were 90.47%, 81.58%, 84.32%, and 84.83%, respectively [110].


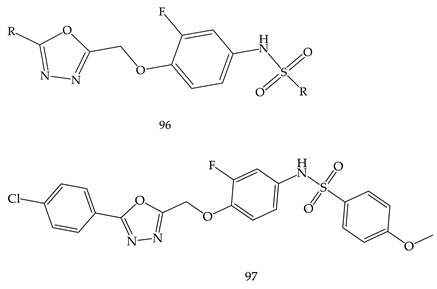


M. M. Gamal El-Din et al. synthesized a series of diarylamides and diarylureas containing 1,3,4-oxadiazole moiety (**98**) and performed their antiproliferative activities against a panel of 58 cell lines of nine different cancer types at the NCI, compared with sorafenib as a reference compound. Among of all derivatives, Compound (1-(3,5-bis(trifluoromethyl)phenyl)-3-(4-((5-(4-chlorophenyl)-1,3,4-oxadiazol-2-yl)methoxy)-3 fluorophenyl) urea) (**99**) showed the highest sub micromolar IC_50_ values of 0.67, 0.80, and 0.87 µM against PC-3 prostate cancer cell line, HCT-116 colon cancer cell line, and ACHN renal cancer cell line, respectively, as shown in Table 1 [111].


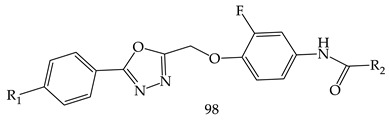
 
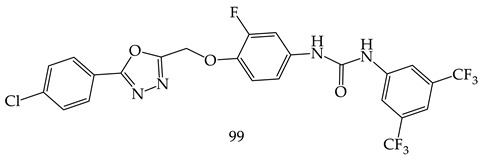


Ahsan et al. synthesized new analogues of the 5-substituted-N-aryl-1,3,4-oxadiazol-2-amine (**100**), which were synthesized and evaluated for anticancer activity as per the guidelines of the NCI, USA, on leukemia, the central nervous system (CNS), ovarian, melanoma, lung, colon, renal, prostate, and breast cancer cell lines [112].


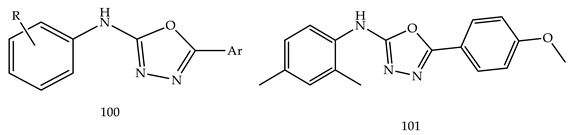


The N-(2,4-Dimethylphenyl)-5-(4-methoxyphenyl)-1,3,4-oxadiazol-2-amine (**101**) displayed the most sensitive action on K-562 (leukemia), MDA-MB-435 (melanoma), HCT-15 (colon cancer) cell lines, and T-47D (breast cancer) with a GP of 18.22, 15.43, 39.77, and 34.27, respectively (Table 2). The maximum growth percent on MDA-MB-435 (melanoma) cell line (GP ¼ 6.82) by Compound N-(2,4-dimethylphenyl)-5-(4-hydroxyphenyl)-1,3,4-oxadiazol-2-amine (**101**), with maximum mean growth percent (GP), was 62.6.

Various 2-naphthalen-1-ylmethyl-1-(5-substituted phenyl-[1,3,4]oxadiazol-2-ylmethyl)-1H-benzimidazole (**102**) and 2-(2-naphthalenyloxymethyl)-1-(5-substituted phenyl-[1,3,4]oxadiazol-2-ylmethyl)-1H-benzimidazole (**103**) were prepared by Salahuddin et al. These reactions were completed by using chloramine-T and phosphorous oxychloride as a catalyst. All synthesized derivatives were screened for their in vitro anticancer effect by NCI at a single high dose (10^−5^ M). Among them all, 2-naphthalen-1-ylmethyl-1-[5-(4-nitro-phenyl)-[1,3,4]oxadiazol-2-ylmethyl]-1H-benzimidazole (**104**) showed prominent activity against MDA-MB-468 (breast cancer) and SK-MEL-28 (melanoma) (GP = 36.23 and 47.56, respectively) [113].


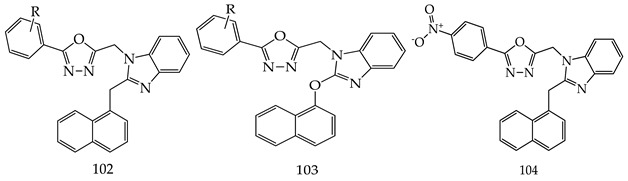


Ahsan et al. prepared a novel series of 2-(4-chlorophenyl)-5-aryl-1,3,4-oxadiazole derivatives (**105**) and reported their molecular docking study of anticancer activity. Out of all synthesized derivatives, two compounds, i.e., 2-(4-chlorophenyl)-5-(4-fluorophenyl)-1,3,4-oxadiazole (**106**) and 2-(4-chlorophenyl)-5-(4-methoxyphenyl)-1,3,4-oxadiazole (**107**) showed better growth percent (GP) against various cell lines(SF-295-CNS cancer, MCF7; breast cancer, PC-3; prostate cancer, SR; leukemia), i.e., 98.74 and 95.37, respectively, in 1 dose 10^−5^ M conc [114].


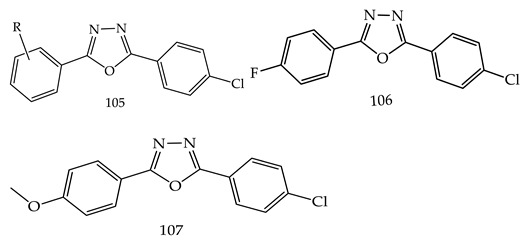


Various 1,3,4-oxadiazole derivatives were synthesized through Mannich reactions and were evaluated for their anticancer activity against tumor cell lines (4T1-mammary carcinoma and CT26.WT-colon cancer cell line) by MTT assay. Among all of the synthesized derivatives containing 1,3,4-oxadiazole, the phenyl and pyridine rings were the most active in the cytotoxic assay. The two most active alkylated Compounds, 3-[(4-dodecylpiperazin-1-yl) methyl]5-phenyl-1,3,4-oxadiazole-2(3H)-thione (**108**) and3-[(4-dodecylpiperazin-1-yl)methyl]-5-(4-methoxyphenyl)-1,3,4-oxadiazole-2(3H)-thione (**109**) have been shown to be capable to induce apoptosis with 1.6 ± 0.3 and 6.3 ± 0.1 and 24.4 ± 2.4 and 1.6 ± 0.7 (IC_50_ (µM± SD)), respectively, against 4T1-mammary carcinoma and CT26.WT-colon cancer cell line [115].


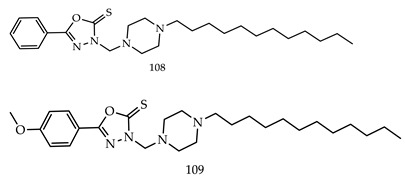


R.Ramesh Raju et al. designed and synthesized novel 2,5-bis(indolyl)-1,3,4-oxadiazoles derivatives and evaluated them for their cytotoxicity against four cancer cell lines, namely, A549, MDA-MB-231, MCF-7, and HeLa, using a MTT-reduced assay. Among all the novel derivatives, Compound 3-[5-(5-bromo-1-methyl-1H-indol-3-yl)-1,3,4-oxadiazol-2-yl]-5-methoxy-1-methyl-1H-indole (**110**) and 1-methyl-3-[5-(5-nitro-1H-indol-3-yl)-1,3,4-oxadiazol-2-yl]-1H-indole (**111**) have shown potent anticancer activities against MCF-7 breast cancer cell line with IC_50_ values of 1.8 ± 0.9 μM and 2.6 ± 0.89 μM, respectively [116].


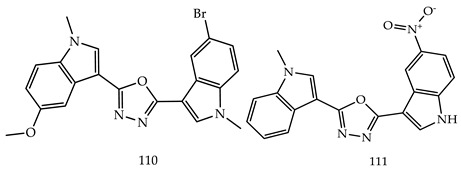


Samar H. Abbas et al. designed and synthesized various novel oxadiazole-chalcone hybrids as EGFR, Src, and IL-6n inhibitors. Compound (**112**) showed the strongest cytotoxic potential against leukemia cell lines K-562, Jurkat, and KG-1a with IC_50_ values of 1.95, 2.36, and 3.45 times μM, respectively. The synthesized compounds also inhibited EGFR, Src, and IL-6. Compound N-(4-(3-(4-Methoxyphenyl) acryloyl) phenyl)-2-((5-(3,4,5-trimethoxy-phenyl)-1,3,4-oxadiazol-2-yl) thio)acetamide (**112**) was the most effective for inhibiting EGFR (IC_50_ = 0.24 μM), Src (IC_50_ = 0.96 μM), and IL-6 (% of control = 20%) at a single concentration (10^−5^ M) [117].


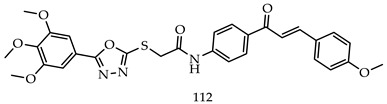


Another three novel series of 2-substituted-5-(4-pyridyl)-1,3,4-oxadiazoles (**113**), 2-substituted-5-(3-pyridyl)-1,3,4-oxadiazoles (**114**) and 2-substituted-5-(phenyl or 4-chlorophenyl-1,3,4-oxadiazoles) (**115**) were designed, synthesized, and evaluated for anticancer activity using a MTT cytotoxicity assay by Reem K. Arafa et al. The three compounds among all derivatives, 2-(2-(4-Nitrobenzylidene)hydrazinyl)-5-(pyridin-4-yl)-1,3,4-oxadiazole (**116**), ethyl N-(5-(pyridin-3-yl)-1,3,4-oxadiazol-2-yl)formimidate (**117**) and N-(4-Chlorobenzylidene)-5-(pyridin-3-yl)-1,3,4-oxadiazol-2-amine (**118**), have shown promising cytotoxic activity, which is shown in Table 3. Out of these three compounds (**117**) (IC_50_-0.2757357 µM) showed nearly double the potency as compared to erlotinib(IC_50_-0.41785 µM), which is the standard drug [118].


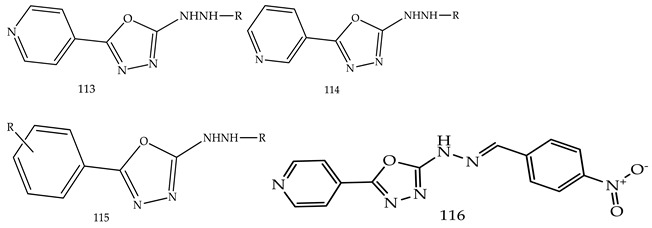
 
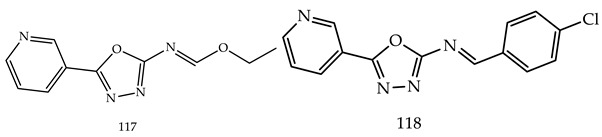


Ping Gonga et al. designed and synthesized novel 1,3,4-oxadiazol acetamide moiety containing 6,7-disubstituted quinoline derivatives (**119**) and evaluated compounds for their anticancer activity via the Axl kinase inhibitory effect. Most of the derivatives had shown moderate to excellent potency, among all the compounds (**119**), (**120**), and (**121**) exhibited excellent Axl enzymatic potency, but among these three selective derivatives Compound N-(3-fluoro-4-((6-methoxy-7-(3-(piperidin-1-yl)propoxy)quinolin-4-yl)oxy)phenyl)-1-(5-(4-fluorophenyl)-1,3,4-oxadiazol-2-yl) cyclopropane-1-carboxamide (**122**) was found to be the most promising as an Axl kinase inhibitor (IC_50_ = 0.010 µM), which shows remarkable cytotoxicity against A549, HT-29, PC-3, MCF-7, H1975, and MDA-MB-231 cell lines, as shown in Table 4 [119].


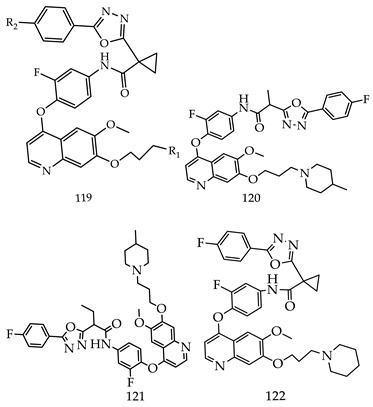


Dhawan S et al. synthesized coumarin conjugated S-alkylated and S-benzylated-1,3,4-oxadiazole derivatives and evaluated them against MDA-MB-231 and MCF-7 breast cancer cell lines. Among the synthesized derivatives, 7-((5-((2,4-Dichlorobenzyl)thio)-1,3,4-oxadiazol-2-yl) methoxy)-4,5-dimethyl-2H-chromen-2-one (**123**) (IC_50_ value less than 5 μM)was found to inhibit the growth of tamoxifen-resistant breast cancer cell lines and was 1.4 times more active against MCF-7 compared to tamoxifen (IC_50_ value = 7.09 μM). A docking study has demonstrated a hydrophobic interaction between Ala45, Leu79, Leu86, and Leu220, with a substituted phenyl ring and coumarin moiety in Compound (**123**) (bond distances = 3.75, 3.64, 3.70, and 3.82Å, respectively) [120].


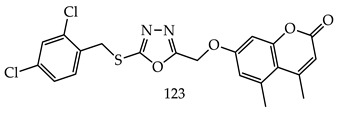


Another novel series of 2,5-disubstituted-1,3,4-oxadiazole derivatives (**124**) were synthesized and screened by Vasanth Raj et al. for all derivatives against human hepatocellular carcinoma cells HepG2 and human breast cancer cells lines MCF-7. Among the synthesized derivatives, 1-[2-(2-Chlorophenyl)-5-(pyridin-4-yl)-1,3,4-oxadiazol-3-(2H)-yl] ethanone (**125**) was found to be the most chemotherapeutic and with cytotoxic action. It was due to the stimulation of the p53 mediated intrinsic pathway of tested cancerous cell lines in HepG2 (human liver cancer cell line) with the highest IC_50_ value [121].


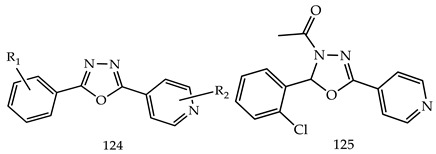


Ewieset al. synthesized novel α-amino phosphonate oxadiazoles derivatives and evaluated them against colon (HCT116), breast (MCF7), and liver (HepG2) human cancer cell lines by standard MTT assay, with doxorubicin as a reference drug. Among the synthesized derivatives, Compounds diethyl (naphthalen-2-yl(2-(2-((5-(pyridin-4-yl)-1,3,4-oxadiazol-2-yl)thio)acetyl) hydrazinyl)methyl)phosphonate (**126**) and diethyl ((2-chlorophenyl)(2-(2-((5-(pyridin-4-yl)-1,3,4-oxadiazol-2-yl)thio)acetyl) hydrazinyl)methyl)phosphonate (**127**) have shown good antiproliferative activities (IC_50_ (µM) ± SD = 9.5 ± 1.5 and 9.2 ± 1.4, respectively,) against HCT116 tumor cells, comparable to doxorubicin (IC_50_ (µM) ± SD = 9.4 ± 3.9) with low cytotoxicity towards normal fetal colon cell (FHC) [122].


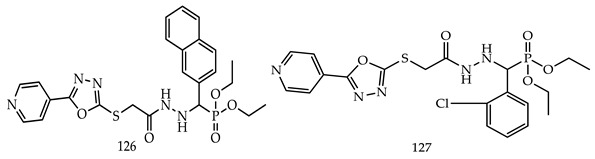


A new series of substituted phenyl[(5-benzyl-1,3,4-oxadiazol-2-yl)sulfanyl]acetates/acetamides (**128**) have been synthesized and evaluated for their inhibitory activity against human alkaline phosphatase (ALP) enzymes. Based on alkaline phosphate inhibitory kinetics, compound 2-(5-benzyl-1,3,4-oxadiazol-2-yl)thio)-N-(3-(trifluoromethyl) phenyl)acetamide (**129**) showed potential anticancer activity, with IC_50_ value 0.420 ± 0.012 μM as comparison to whereas the standard IC_50_ (2.80 μM). Studies of molecular docking against alkaline phosphatase enzyme (1EW2) showed that Compound (**129**) had excellent binding affinity with a binding energy value of −7.90 kcal/mol compared to other derivatives [123].


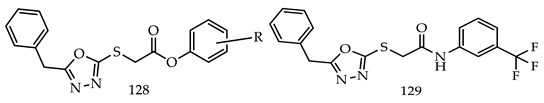


Ismail celiket al. designed and synthesized a number of novel 1,3,4-oxadiazole-benzimidazole derivatives. MTT assays were used to evaluate their cytotoxicity against five cancer cell lines, i.e., A549, MCF-7, C6, HepG2, and HeLa. Out of theses cancer cell lines, derivatives (**130**) (IC_50_ = 6.554 ± 0.287 μM) and (**131**) (IC_50_ = 5.132 ± 0.211 μM) were found to have satisfactory potencies and higher anticancer activities, as compare to doxorubicin (IC_50_ = 10.525 ± 0.472 μM) against only MCF-7. An in vitro, aromatase (ARO) enzyme inhibition assay was also performed. Additionally, molecular docking studies were conducted to detect their binding sites and types of interactions with aromatases. Compounds (**130**) and (**131**) were also investigated via molecular dynamic simulations for its possible binding mode to CYP19A1 and it was found that both derivatives had great potential as compared to others [124].


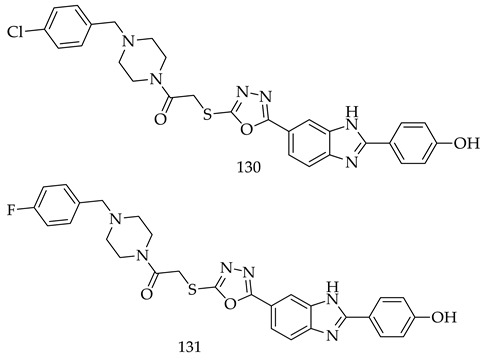


In order to evaluate the cytotoxicity of this novel class of 1,3,4-oxadiazole hybrids (**16**), novel naproxen-based naproxen derivatives were synthesized and screened for cytotoxicity against EGFR by Mohammad M Alam and colleagues. There were several compounds synthesized, but 4-((5-((S)-1-(2-methoxynaphthalen-6-yl)ethyl)-1,3,4-oxadiazol2-ylthio)methyl)-1H-1,2,3-triazol-1-yl)phenol (**132**) showed the greatest potency against MCF-7 and HepG2 cancer cells, and was equipotent to doxorubicin (IC_50_ 1.62 µg/mL) towards HepG2. A further study showed that Compound (**132**) inhibited the EGFR kinase with an IC_50_ of 0.41 m, which was greater than the IC_50_ of the standard drug erlotinib (IC_50_ = 0.30 m). Cells from MCF7, HePG2, and HCT 116 are affected by the active compound in a high percentage. Biological data were also supported by docking studies, DFT, and MEP. By bind ring to adenosine triphosphate (ATP), these naproxen hybrids inhibit EGFR kinase by competitively inhibiting tyrosine kinase [125].


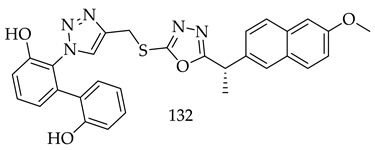


Twenty-seven derivatives of 5-substituted 2-amino-1,3,4-oxadiazole and 2-amino-1,3,4-thiadiazoles were synthesized. A diffusion method was used to determine the antibacterial and antifungal activities, while MTT assay was used to determine the anticancer activities. The researchers found that conjugate (**133**) displayed remarkable cytotoxic activity against HepG2 cell line (IC_50_ = 8.6 μM), which is comparable to the activity of paclitaxel, and is non-toxic on LLC-PK1 normal cell line. According to the structure-activity relationship and the molecular docking study of the synthesized compound, ethoxy, halogen, and nitro derivatives possess significant antimicrobial and cytotoxic properties [126].


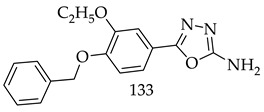


M. Strzelecka and colleagues synthesized and characterized a new series of N-Mannich base-type hybrid compounds containing oxadiazole ring, 1,3,4-oxadiazole ring, and 4,6-dimethylpyridine ring., including the A375, C32, SNB-19, MCF-7/WT, and MCF-7/DX. Researchers were explored all compounds for anticancer properties, and revealed that, two Compounds (**134**) (IC_50_ = 80.79 µM) and (**135**) (IC_50_ = 202.47 ± 10.12 µM) were found with promising anticancer potentials, which was also examined for their anticancer activity on melanoma cells (A375 and C32) and human normal cells (keratinocytes). As per the MTT assay, among all derivatives, Compound 134 was most significantly cytotoxic to A375 cells (IC_50_ = 80.79 µM) as comparison to cisplatin as a standard drug [127].


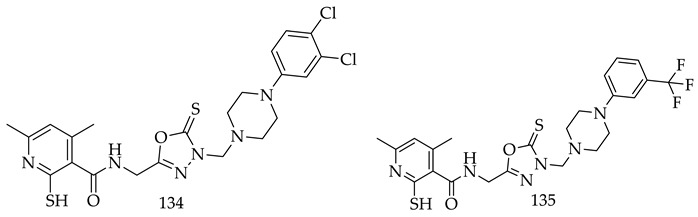


Similarly, another antiproliferative study via MTT assay was performed by M. M alam and his colleagues by synthesizing a novel series of 1,2,3-triazole-incorporated thymol-1,3,4-oxadiazole conjugates (**136**). They tested all 14 derivatives against three different cancer cell lines, i.e., MCF-7, HCT-116, and HepG2, and found derivative (**137**) the most potent against all cell lines. Compound (**137**) (2-(4-((5-((2-isopropyl-5-methylphenoxy) methyl)-1,3,4-oxadiazol-2-ylthio) methyl)-1H-1,2,3-triazol-1-yl)phenol) had shown significant potential against all three tested cell lines, MCF-7 (IC_50_ 1.1 µM), HCT-116 (IC_50_ 2.6 µM), and HepG2 (IC_50_ 1.4 µM), as comparison to reference drug doxorubicin MCF-7 (IC_50_ = 1.2 µM), HCT-116 (IC_50_ = 2.5 µM), HepG2 (IC_50_ = 1.8 µM), 5-fluorouracil MCF-7 (IC_50_ = 18.74 µM), HCT-116 (IC_50_ = 30.68 µM), and HepG2 (IC_50_ = 28.65 µM) [128].


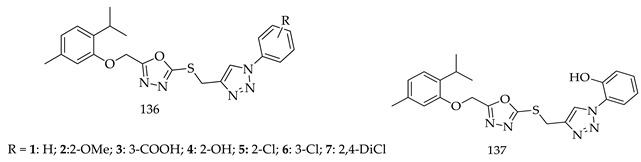


The structure-activity relationships (**138**) of a synthesized novel series of 1,2,3-triazole-incorporated thymol-1,3,4-oxadiazole conjugates revealed enhanced selectivity towards better lead derivatives, for further research:

(i) Compared to meta- and para-substituted derivatives, ortho substituted derivatives have remarkable potential. Therefore, derivatives 7, 9, and 10, bearing (2-OMe), (2-OH), and (2-Cl), respectively, on the phenyl ring showed the highest activity as comparison to derivatives 8 (3-COOH), 11 (3-Cl), and 13 (4-Br).
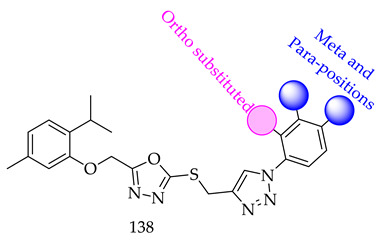
(ii) In comparison with monosubstituted derivatives (**139**), disubstituted derivatives (**140**) have decreased cytotoxicity.

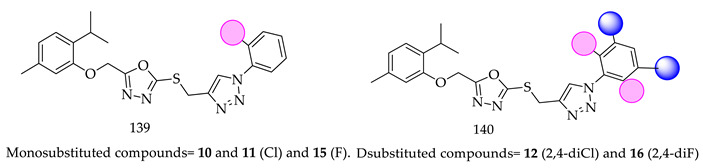


Ibrahim H. Eissa et al. synthesized a series of 14 compounds of 1,3,4-oxadiazole-naphthalene hybrids (141) and tested their cytotoxicity against MCF-7 (human breast cancer cell line) and HepG2 (human hepatocellular carcinoma cell line) via MTTS assay. 06 derivatives (**142**, **143**, **144**, **145**, **146**, and **147**) showed the highest potential among the 14 synthesized derivatives and their efficacy against VEGFR-2 also evaluated for further studies.


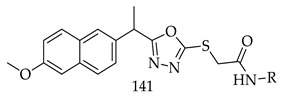
 
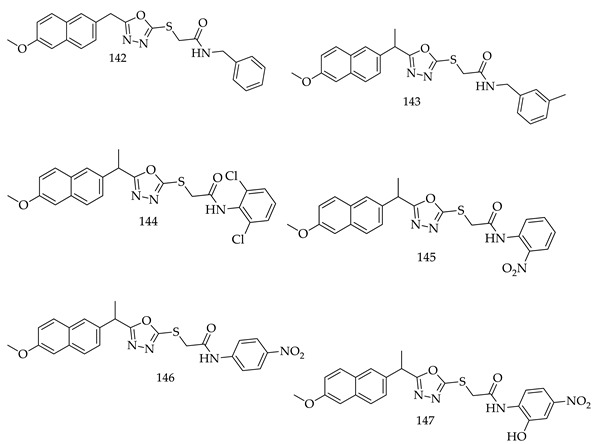


A good antiproliferative effect was observed for Compound (**142**) against both cell lines and inhibitory activity was demonstrated against VEGFR-2. It also induced 22.86% apoptosis compared to 0.51% apoptosis in control (HepG2) cells [129].

A new series of furo[2,3-d]pyrimidine–1,3,4-oxadiazole hybrid derivatives were synthesized and evaluated for their cytotoxic activity in four human cancer cell lines: fibrosarcoma (HT-1080), breast (MCF-7 and MDA-MB-231), and lung carcinoma (A549). Data showed that Compound 8f exhibits moderate cytotoxicity, with IC_50_ values ranging from 13.89 to 19.43 μM. Besides, Compound (**148**) induced apoptosis through caspase 3/7 activation, cell death independently of the mitochondrial pathway, and cell cycle arrest in the S phase for HT1080 cells and the G1/M phase for A549 cells [130].


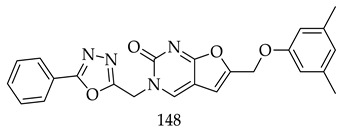


A series of novel 1,3,4-oxadiazole derivatives with substituted phenyl ring were designed, synthesized and evaluated for cytotoxicity by the MTT method against two breast cancer cell lines (MCF-7 and MDA-MB-231). Further, the results of TP assay identified that 1,3,4-oxadiazole molecules displayed anticancer activity partially by the inhibition of phosphorylation of thymidine. The TP assay identified (**149**) and (**150**) as potential inhibitors with anticancer activity against both the cell lines [131].


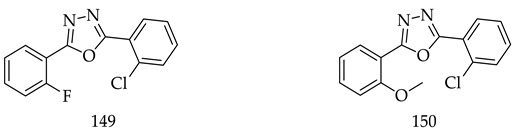


A.E Mansouri and his collaborators designed and synthesized a series of homonucleosides containing theophylline,1,3,4-oxadiazole derivatives (**151**). Each compound was tested their in vitro cytotoxicity against four cancer cell lines, including HT-1080 (fibrosarcoma), MCF-7 and MDA-MB-221 (breast), and A-549 (lung carcinoma). All synthesized derivatives had shown mild to moderate growth inhibition against all the cell lines, except Compound (**152**), which has shown significant inhibition percentage (78%) and IC_50_ values of 17.08 ± 0.97 µM against fibrosarcoma. Further, molecular docking studies confirmed that Compound (**152**) causes apoptosis by activating caspase-3 (caspase protein) by forming hydrogen bonds and hydrophobic interactions [132].


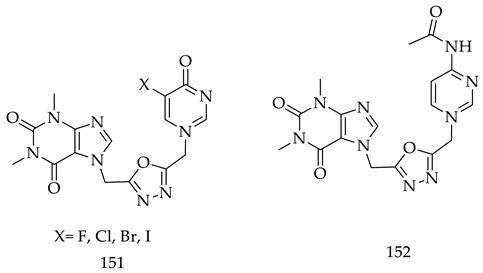


Merccaptoacetamide-linked pyrimidine-1,3,4-oxadiazole hybrids (**153**) were designed and synthesized by Arbaz Sujat Shaikh and colleagues. The novel pyrimidine-1,3,4-oxadiazole hybrids were evaluated for their in vitro cytotoxic potential against 04 cancer cell lines, i.e., lung cancer cells (A549), prostate cancer cells (PC-3, DU-145), and human embryonic kidney cells (HEK) [133].


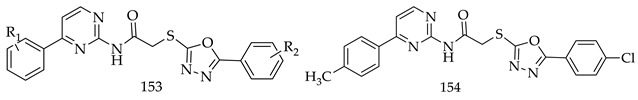


**Figure 23:**
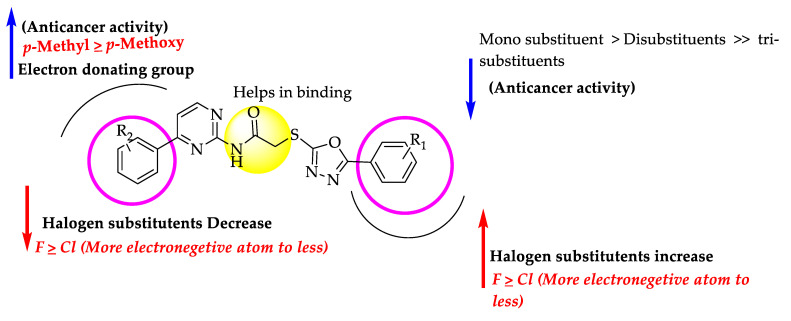
The structure-activity relationship of pyrimidine-1,3,4-oxadiazole analogues.

Compound (**154**) (2-((5-(4-chlorophenyl)-1,3,4-oxadiazol-2-yl)thio)-N-(4-(p-tolyl)pyrimidin-2-yl)acetamide) demonstrated greatest potency against A549 cells, with an IC_50_ of 3.8 ± 0.02 μM. For the noncancerous cell line (HEK), Compound (**154**) demonstrated 25-fold greater affinity. The structure-activity relationship also revealed that halogen substituents and EDGs have a great impact of anticancer potential of the synthesized derivatives (Figure 23).

Further, a mixture of molecular modeling and target-based assay studies proved that Compound (**154**) was well accommodated at the active site of the DNA topoisomerase II complex, and exhibited favorable physiochemical and ADME/T properties in silico. Similarly, a series of imidazo[1,2-a]pyridine-oxadiazole hybrids (**155**) was synthesized and tested for their in vitro anticancer potential against lung cancer cells (A549) and prostate cancer cells (PC-3, DU-145) by Dilep Kumar Sigalapalli and his team.


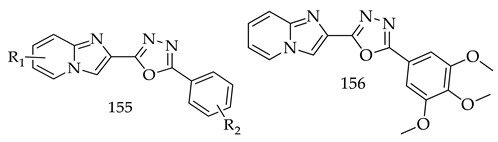


As a result of screening the all-synthesized compounds, derivative (**156**) was found to be the most potent on A549 cells with an IC_50_ value of 2.8 ± 0.02 μM, as a comparison to podophyllotoxin (standard drug), with an IC_50_ value of 0.09 ± 0.01. The effect of derivative (**156**) on different phases of the cell cycle was also determined by annexin-v/PI dual staining (economical dye for apoptotic cell investigation) and it as found that Compound (**156**) induces apoptosis in A549 cells. The molecular modeling studies also revealed that derivative (**156**) had a significant affinity towards the tubulin receptor, binding with significant physico-chemical properties. Structure-activity relationships also revealed that halogen substituents and EDGs have an important impact on the anticancer potential of synthesized derivatives. Halogen atoms at para position on phenyl rings linked to 1,3,4-oxadiazole decreases anticancer potential, and EDGs increases the anticancer potential of designed derivatives [134].

A new series of 1,2,3-triazole/thioacetamide linked benzimidazole-based 1,3,4-oxadiazole derivatives (**157**) has been prepared by Syed Nazreen and colleagues. Cell lines MDA-MB-231, SKOV3, and A549, were studied for the cytotoxicity of the synthesized conjugates cytotoxicity via MTTS assay, and EGFR inhibition was investigated.


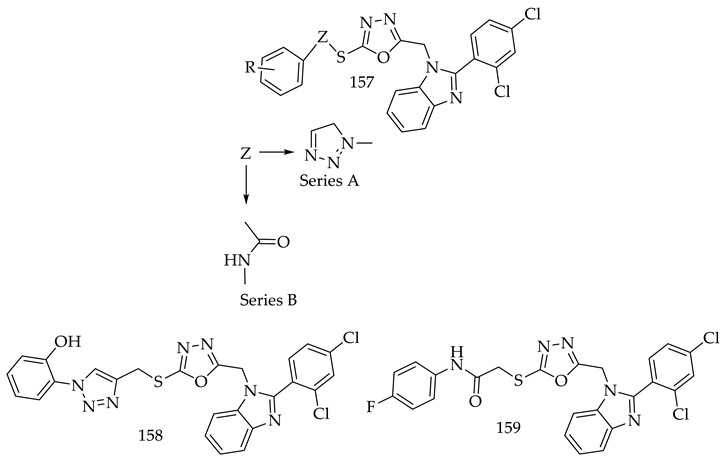


Compounds (**158**) and (**159**) showed the most promising cell cycle distribution, which provided insight into their intracellular mechanism of action. The A549 cells treated with Compound (**157**) exhibited a decrease in S phase distribution and an increase in G1 and G2 phases, while Compound (**158**) exhibited an increase in G1 phase distribution and a decrease in S phase distribution. Compound (**158**) increased the number of cells in the G1 phase in MDA-MB-231 breast cells, while decreasing the number of cells in the G2 phase. SKOV3 cells showed an increase in S phase cell distribution, but a decrease in G1 phase cell distribution when treated with Compound (**158**). In non-small cell lung cancer, erlotinib impairs the G0/G1 phase; in hepatocellular carcinoma, it impairs the G1/S checkpoint; and in esophageal cancer, it impairs the G1/G0 phase. The S, G1, and G2 phases of the cell cycle are arrested by Compounds (**158**) and (**159**) [135].

## 8. Patents

The research and development of oxadiazole-based drugs has gained popularity in the last decades due to their properties such as low toxicity, good efficacy and safety, fine biochemical diversity. 1,3,4 oxadiazole and its derivatives have long been known for their abilities to inhibit cancer-causing cells, growth factors, kinases, and a number of other biological enzymes. As shown in Figure 7, 1,3,4 oxadiazole rings have proven their potential in numerous therapeutic drugs. In this way, the 1,3,4 oxadiazole ring played an important role in the development of a variety of anticancer lead structures. As a continuation of the 1,3,4 oxadiazole ring potential, a large number of anticancer patents have been filed/published.


**S. No**

**Patent No**

**Publication Date**

**Title**

**References**

**1.**
US306523820 November 1962Production of 1,3,4-oxdiazoles[136]
**2.**
US288339121 April 1959Method of making 2-amino-5-substituted-1,3,4-oxadiazoles[137]
**3.**
87963205 August 20141,3,4-Oxadiazole-2-carboxamide compound[138]
**4.**
884672830 September 2014Sphingosine 1-phosphate (S1P) receptor modulators[139]
**5.**
884672930 September 20142-thio-1,3,4-oxadiazoles azetidine derivatives as sphingosine-1 phosphate receptors modulators[140]
**6.**
WO03330112 March 20151,3,4-Oxadiazole And 1,3,4-Thiadiazole Derivatives as Immunomodulators[141]
**7.**
873543327 May 2014Sphingosine1-phosphate (S1P) receptor modulators[142]
**8.**
85247513 September 20134-Oxadiazol-2-YL-indazoles as inhibitors of P_13_ kinases[143]

## 9. Conclusions

In order to treat cancer more effectively, and with a low-level of toxicity, scientists are developing new anticancer agents. The treatment of drug-resistant cancer is limited due to the lack of anticancer drugs. Therefore, new antitumor drugs are urgently needed. Thus, the 1,3,4-oxadiazole scaffold remains an important target in modern medicinal chemistry for the discovery of new therapeutic leads. Further, 1,3,4-oxadiazole consists of two atoms of nitrogen interconnected with two atoms of carbon that are electron-deficient, and two-electron pairs with an active oxygen atom attached to them. This review has compiled numerous synthetic chemical schemes over the past decades, and various anticancer activities of 1,3,4-oxadiazole derivatives were described. Aside from the molecular targets and pathways involved in carcinogenesis, the review explored mechanisms of action (MOAs), docking, and patents granted.

The docking of 1,3,4-oxadiazoles as antiproliferative agents is a very significant discovery in medical research. Molecular docking is important for designing specific drug delivery systems. Molecular docking study plays a significant role in the anticancer study of 1,3,4-oxadiazoles derivatives. In this review article, we summarized how molecular docking study helps to provide information in the binding of 1,3,4-oxadiazoles derivatives with different protein sites for their anticancer potentials.

This structure-activity relationship study contributes to the development of new 1,3,4-oxadiazole-heterocycle hybrids with low toxicity to healthy cells, due to the presence of more than one pharmacophore in a hybrid. During the last 10 years, hybrids of 1,3,4-oxadiazoles and heterocycles have shown huge anticancer potential due to extensive study on their structure modifications. A clear idea of how nontoxic, effective, anticancer drugs can be developed has also been provided through a review of the SAR and mechanisms of action of the hybrids. Therefore, the study of the 1,3,4-oxadiazole scaffold as anticancer agent has been well demonstrated and identify a possible pharmacophore that can be combined with existing compounds to boost anticancer activity. Several researchers have been interested in exploring the many potential applications of oxadiazole derivatives due to their broad pharmacological profiles. Future therapeutic molecules are likely to contain an oxadiazole motif, which helps in the development of novel medicines for the treatment of patients suffering from various tumors.

## Figures and Tables

**Table 1:** The anticancer activity of 1,3,4-oxadiazole Compound (**99**).

Cell Line	Type of Cancer (s)	IC_50_ (µM)	IC_50_ (µM) Sorafenib
PC-3	Prostate	0.67	1.99
HCT-116	Colon	0.80	1.58
ACHN	Renal	0.87	2.51

**Table 2:** The anticancer activity of N-(2,4-dimethylphenyl)-5-(4-hydroxyphenyl)-1,3,4-oxadiazol-2-amine against various cell lines.

Cell Lines	K-562 (Leukemia)	MDA-MB-435 (Melanoma)	HCT-15 (Colon Cancer)	T-47D (Breast Cancer)
Growth percent (GP)	18.22	15.43	39.77	34.27

**Table 3:** The EGFR kinase inhibitory activity of substituted 1,3,4-oxadiazoles.

Compd. No.	IC_50_ (µM)
116	0.4123 ± 0.022
117	0.2757 ± 0.013
118	1.1289 ± 0.045
Erlotinib (Standard)	0.4178 ± 0.014

**Table 4:** The inhibitory activities of 1,3,4-oxadiazol acetamide moiety containing6,7-disubstituted quinoline derivatives against Axl.

Comp. No.	IC_50_ on Axl (μM)
120	0.036
121	0.077
122	0.010
Foretinib (Standard)	0.014

## Data Availability

Data sharing is not applicable to this article. It is the author’s opinion that the study has no DOI, since no new data were created or analyzed in this review article study.
